# MicroRNA-655-3p and microRNA-497-5p inhibit cell proliferation in cultured human lip cells through the regulation of genes related to human cleft lip

**DOI:** 10.1186/s12920-019-0535-2

**Published:** 2019-05-23

**Authors:** Mona Gajera, Neha Desai, Akiko Suzuki, Aimin Li, Musi Zhang, Goo Jun, Peilin Jia, Zhongming Zhao, Junichi Iwata

**Affiliations:** 1 Department of Diagnostic & Biomedical Sciences, School of Dentistry, The University of Texas Health Science Center at Houston, Houston, TX USA; 2 Center for Craniofacial Research, The University of Texas Health Science Center at Houston, Houston, TX USA; 3 Center for Precision Health, School of Biomedical Informatics, The University of Texas Health Science Center at Houston, Houston, TX USA; 4 Department of Epidemiology, Human Genetics & Environmental Sciences, School of Public Health, The University of Texas Health Science Center at Houston, Houston, TX USA; 5 MD Anderson Cancer Center UTHealth Graduate School of Biomedical Sciences, Houston, TX USA

**Keywords:** Cleft lip, Gene mutation, MicroRNA, Systematic review, Bioinformatics analysis

## Abstract

**Background:**

The etiology of cleft lip with or without cleft palate (CL/P), a common congenital birth defect, is complex and involves the contribution of genetic and environmental factors. Although many candidate genes have been identified, the regulation and interaction of these genes in CL/P remain unclear. In addition, the contribution of microRNAs (miRNAs), non-coding RNAs that regulate the expression of multiple genes, to the etiology of CL/P is largely unknown.

**Methods:**

To identify the signatures of causative biological pathways for human CL/P, we conducted a systematic literature review for human CL/P candidate genes and subsequent bioinformatics analyses. Functional enrichment analyses of the candidate CL/P genes were conducted using the pathway databases GO and KEGG. The miRNA-mediated post-transcriptional regulation of the CL/P candidate genes was analyzed with miRanda, PITA, and TargetScan, and miRTarbase. Genotype-phenotype association analysis was conducted using GWAS. The functional significance of the candidate miRNAs was evaluated experimentally in cell proliferation and target gene regulation assays in human lip fibroblasts.

**Results:**

Through an extensive search of the main biomedical databases, we mined 177 genes with mutations or association/linkage reported in individuals with CL/P, and considered them as candidate genes for human CL/P. The genotype-phenotype association study revealed that mutations in 12 genes (*ABCA4*, *ADAM3A*, *FOXE1*, *IRF6*, *MSX2*, *MTHFR*, *NTN1*, *PAX7*, *TP63*, *TPM1*, *VAX1*, and *WNT9B*) were significantly associated with CL/P. In addition, our bioinformatics analysis predicted 16 microRNAs (miRNAs) to be post-transcriptional regulators of CL/P genes. To validate the bioinformatics results, the top six candidate miRNAs (miR-124-3p, miR-369-3p, miR-374a-5p, miR-374b-5p, miR-497-5p, and miR-655-3p) were evaluated by cell proliferation/survival assays and miRNA-gene regulation assays in cultured human lip fibroblasts. We found that miR-497-5p and miR-655-3p significantly suppressed cell proliferation in these cells. Furthermore, the expression of the predicted miRNA-target genes was significantly downregulated by either miR-497-5p or miR-655-3p mimic.

**Conclusion:**

Expression of miR-497-5p and miR-655-3p suppresses cell proliferation through the regulation of human CL/P-candidate genes. This study provides insights into the role of miRNAs in the etiology of CL/P and suggests possible strategies for the diagnosis of CL/P.

**Electronic supplementary material:**

The online version of this article (10.1186/s12920-019-0535-2) contains supplementary material, which is available to authorized users.

## Background

Cleft lip with or without cleft palate (CL/P) is a common congenital malformation affecting speech, hearing, feeding, among others functions [1]. Individuals with CL/P require a comprehensive treatment, including multiple plastic and maxillofacial surgeries from birth to adulthood, and speech therapy [2]. It has an average worldwide birth prevalence of 1 in 1000, ranging from 1 in 500 in the Asian population to 1 in 2500 in the African population, with wide variability per geographic origin, ethnicity, and socioeconomic status [3, 4]. The complexity of its etiology seems to result from various genetic and environmental risk factors along with gene- environment interactions (e.g., approximately 70% cases are non-syndromic) [5]. Genome-wide association studies (GWAS) and linkage studies have identified genetic susceptibility to CL/P and differences in such susceptibility in various populations and ethnic groups [6]. However, as seen in many complex diseases or traits, most of the mutations and loci identified from such studies are in the noncoding genomic regions and do not have specific functional annotations [7, 8]. Although much progress has been made in identifying genes whose mutations are associated with CL/P, little is known about the mechanisms by which environmental and epigenetic factors adversely influence gene expression during lip development. Recent studies indicate that environmental factors contribute to changes in phenotype or gene expression at post-transcriptional level through the regulation of noncoding RNAs, including microRNAs (miRNAs) [9]. miRNAs are short (~ 22 nucleotides) noncoding RNA molecules that regulate gene expression at the post- transcriptional level, and they fine-tune the expression of ~ 30% of all mammalian protein-encoding genes [10]. miRNAs were first reported in mammalian systems in 2001 [11], but the latest release of the miRNA database [miRBase (ver. 20): more than 24,000 miRs annotated] highlights the rapid growth of this field of research; however, the expression patterns and functions of most miRNAs still remain to be discovered. The miRNA-gene regulatory mechanisms contribute to the pathogenesis in various diseases [12, 13]. Nonetheless, a limited number of miRNAs (e.g. miRNA- 140, miRNA-17-92 cluster, miRNA-200b, miRNA-133b) have been reported as miRNAs involved in craniofacial deformities in zebrafish and mouse models, as well as in humans [14]. Therefore, an investigation of the functional regulation, at the level of biological pathways and post- transcriptional regulation mechanism, will improve our understanding of genetic susceptibility to CL/P [7, 15].

In this study, in order to identify signatures of causative pathways in the complex etiology of CL/P, we performed a systematic review and subsequent bioinformatics analysis for CL/P-candidate genes. In addition, we analyzed miRNA-gene regulation by bioinformatics analyses. The function in cell proliferation/survival for six candidate miRNAs was experimentally evaluated by cell proliferation assays. Our findings will help us understand the role of genes and miRNAs that are associated with CL/P, in biological pathways and networks. Such knowledge will provide the basis for the diagnosis, prevention, and treatment of craniofacial anomalies such as CL/P.

## Methods

### Data sources

The publishing guidelines set forth by PRISMA (Preferred Reporting Items for Systematic Reviews and Meta- Analyses) were followed during the literature search and review. The search was conducted using three main literature databases: Medline (Ovid), PubMed (NLM), and Embase (Ovid). In addition, related citations were searched in Scopus (Elsevier, Inc.) to check whether any unique studies were missed from the regular database searches.

### Inclusion and exclusion criteria

The articles meeting the following eligibility criteria were included in our systematic review:

- described genes causing or potentially associated with CL/P in humans;
- referred to studies of either syndromic or non-syndromic CL/P;
- were published as original articles (not as review articles, editorials, dissertations, conference proceedings or comments);
- included cross-sectional, case-control, cohort studies, or clinical trials;
- were published in English.

After screening for articles using the criteria above, we manually excluded those articles in which:

- gene mutations were not described in the original articles;
- CL/P was not specifically described;
- CL/P resulted from environmental factors;
- treatments for CL/P were described;
- were case reports;
- the articles failed to fit in any of the above criteria but did not have CL/P candidate genes or related information.

### Search methods to identify studies

Concepts included in the search were: CL/P, genetics (gene mutations), and humans. A combination of Medical Subject Headings (MeSH) terms and titles, abstracts, and keywords was used to develop the initial Medline search string, and then adapted to search the other databases. The last search date was May 28, 2018.

### Study selection and data collection

All the citations found in the search process were stored in RefWorks (ProQuest) and any duplicates were excluded. Search strategies and results were tracked using the Primary Excel Workbook designed for systematic reviews (http://libguides.sph.uth.tmc.edu/excel_SR_workbook). To check the reliability of study selection between the screeners, Cohen’s Kappa test was performed using a randomly selected sample of 66 citations screened for CL/P-candidate genes by titles and abstracts. After achieving *a* > 90% score for the Cohen’s Kappa, all the titles and abstracts of the articles found through the database search were independently examined by two screeners. The full text of the articles not excluded in the above process was manually reviewed, and all results from the screening were recorded in the Primary Excel Workbook. The data collected were displayed as a descriptive narrative. A codebook for data extraction from eligible articles was developed, as described previously [16]. The data elements extracted for the codebook included citation information, study level information (characteristics and results), and quality level information. The quality assessment of each study identified was performed using the Newcastle- Ottawa Scale (NOS), considering the selection criteria of pre- and post- fortification samples, comparability of these groups, and the ascertainment of either the exposure or outcome. NOS assigns a maximum score of 9 points where studies showing < 5 points have high risk of bias and limitations, with these being excluded from a meta-analysis [17].

### Bioinformatics analysis of CL/P genes

Functional enrichment analyses of the candidate genes were conducted using the pathway databases Gene Ontology (GO) and Kyoto Encyclopedia of Genes and Genomes (KEGG) through tools of the database for annotation, visualization, and integrated discovery (DAVID, version 6.8, http://david.ncifcrf.gov) [18]. The enrichment of CL/P genes in a pathway or GO term was tested by the hypergeometric test implemented in DAVID. The *p*-values were adjusted by false discovery rate (FDR, *q*-value). The significant pathways were obtained by *q* < 0.05 and at least four genes from the list of input genes (CL/P genes) in each pathway included. Hierarchical level 4 was used as the cutoff in order to avoid too general GO terms. The miRNA- mediated post-transcriptional regulation of the CL/P candidate genes was analyzed by using the following method: first, the miRNA-gene pairs were identified from three computationally predicted miRNA-target gene interaction databases, miRanda, PITA, and TargetScan [19–22]; then, the miRNA-gene pairs that were experimentally proofed were selected through miRTarbase analysis [13]; finally, for each miRNA, the enrichment of CL/P candidate genes was examined using Fisher’s Exact Test. To investigate which human phenotype terms are strongly enriched for the candidate genes, the WEB-based Gene SeT AnaLysis Toolkit (WebGestalt) (http://webgestalt.org) was used to perform the overrepresentation enrichment analysis (OEA) using the Human Phenotype Ontology (HPO) annotation database. The top 30 results from the OEA were retrieved on March 15, 2019. The minimum number of genes per category was set to 5 (default). The Benjamini–Hochberg procedure was used for multiple test correction [23]. FDR values were used for all statistical analyses.

### Genotype-phenotype association analysis

Genotype-phenotype association analysis was conducted using GWAS available from dbGaP (dbGaP accession phs000774.v1.p1, https://www.ncbi.nlm.nih.gov/gap). This dbGaP repository includes 11,925 individuals from 4058 families, mostly from trios. Out of 11,925 individuals, 5327 were reported as Caucasians, 2221 as Asians, 473 as Africans, and 3904 were reported as having more than one ancestry. A total of 5008 individuals were reported as of Hispanic ethnicity. Not including the parents, 52% of children were males and 48% were females. Array-based genotypes from Illumina Infinium HumanCore Beadchips, including GWAS markers from HumanCore v1 with additional exome and custom contents from the Center for Craniofacial and Dental Genetics (CCDG) consortium, were analyzed with the PLINK software (version 1.90b) to test the association of each directly genotyped variant in the genes listed through the systematic review. Transmission disequilibrium test (TDT) was applied for maximizing analytical power for trios and for minimizing artifacts from population stratification.

### Cell culture

Human lip fibroblasts were obtained from JCRB Cell Bank (#JCRB9103KD) and cultured in Dulbecco’s Modified Eagle Medium (DMEM) supplemented with 10% fetal bovine serum (FBS), penicillin/streptomycin, and L-glutamine. Human lip fibroblasts were plated onto 96-well cell culture plates at a density of 5000/well and treated with a mimic for either negative control, miR-124-3p, miR- 369-3p, miR- 374a-5p, miR-374b-5p, miR-497-5p, and miR-655-3p (mirVana miRNA mimic, ThermoFisher Scientific) using TransIT-X2 system (Mirus Bio LLC, Madison, WI), following the manufacturer’s protocol (3 pmol mimic and 0.3 μl transfection reagent in 100 μl DMEM per well). Cell proliferation was determined using a cell counting kit 8 (Dojindo Molecular Technologies, Gaithersburg, MD) (*n* = 6 per group).

### Quantitative RT-PCR

Total RNAs were isolated from cultured human lip fibroblasts treated with a mimic for target miRNAs for 1 day (n = 6 per group), using the QIAshredder and RNeasy mini extraction kit (QIAGEN), as previously described [24]. The PCR primers used for quantitative RT-PCR are listed in Additional file 1: Table S1.

### Statistical analysis

Two-tailed student’s *t* tests were applied for the statistical analysis. A *p* value < 0.05 was considered statistically significant. For all graphs, data are represented as mean ± standard deviation (SD).

## Results

### Literature search

**Fig. 1:**
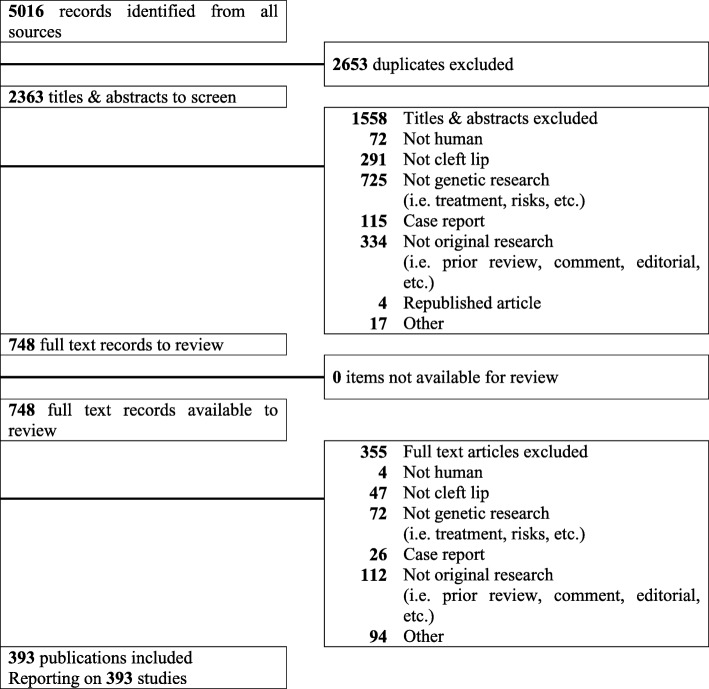
PRISMA flowchart for study selection. A graphical representation of the flow of citations reviewed in the course of the systematic review was provided using a PRISMA flow diagram

Our systematic search identified a total of 5016 publications. After eliminating 2653 duplicates from the list, the remaining 2363 articles were further screened, using the titles and abstracts, independently by the two screeners, which resulted in 1558 publications being further excluded based on reasons such as referring to non-genetic studies and case reports. The remaining 748 articles were further assessed for eligibility through manual full-text review. Through this process, 393 articles satisfying all inclusion criteria were selected while 355 articles were excluded. These selected 393 studies were used for collection of CL/P-candidate genes and in the follow-up analyses (Fig. 1 and Additional file 1: Table S2).

### Summary of human CL/P genes

We identified 172 CL/P-candidate genes from the qualified studies above (Table 1 and Additional file 1: Tables S3–S8). For the bioinformatics analyses, we excluded phenotypic markers and genes with unknown genomic location. Among the CL/P-candidate genes, 10 genes were studied at least five times in different populations: *IRF6* (52 studies, encoding interferon regulatory factor 6, located at genomic locus 1q32.2), *MTHFR* (26 studies, encoding methylenetetrahydrofolate reductase, at 1p36.2), *TGFA* (18 studies, encoding transforming growth factor alpha, at 2q13.3), *MSX1* (25 studies, encoding msh homeobox 1, at 4p16.2), *TGFB3* (16 studies, encoding transforming growth factor beta 3, at 14q24.3), *NECTIN1* (10 studies, encoding Nectin cell adhesion molecule 1, at 11q23.3), *BMP4* (10 studies, encoding bone morphogenetic protein 4, at 14q22.2), *FOXE1* (6 studies, encoding forkhead box E1, at 9q22.33), *BCL3* (6 studies, encoding B-cell CLL/lymphoma 3, at 19q13.32), and *CRISPLD2* (5 studies, encoding cysteine rich secretory protein LCCL domain containing 2, at 16q24.1). Most of the gene mutations (168/177 = 94.9%) were reported only in non-syndromic CL/P while mutations in nine genes (*ADH1C*, *FGFR1*, *IRF6*, *MID1*, *NECTIN1*, *PHF8*, *SOX9*, *TGFA*, and *TP63*) were also reported in syndromic CL/P (9/177 = 5.1%). Several genes were reported only in syndromic cases; *CHD7* (CHARGE syndrome), *CR1* (van der Woude syndrome), *EFNB1* (craniofrontonasal syndrome), *KISS1R* (Kallmann syndrome), *MID1* (Opitz G/BBB syndrome), *REN* (van der Woude syndrome), and *SOX9* (Pierre-Robin syndrome). Mutations in eight genes (*BCL3*, *BMP4*, *IRF6*, *MSX1*, *MTHFR*, *NECTIN1*, *TGFA*, and *TGFB3*) were reported across different ethnic groups such as Caucasians, African Americans, Hispanics, and Asians, including but not limited to Chinese, Japanese, Indian, Turkish, Polish, Finnish, Brazilian, American, and European. By contrast, a total of 75 genes were reported as insignificant in some populations. For example, while mutations in *TGFA* were significant in the Iranian, Korean, Caucasian and Chilean population, they were not significant in the South American, Italian, Malaysian or Indian populations. Mutations in *IRF6* were not significant in some studies, but they were significant in larger studies in various populations (Additional file 1: Tables S3–S8). Recent advances in the discovery of genetic variants at whole genome level and the design of genome-wide approaches enable investigators to identify the involvement of multiple genes and loci in a single study (e.g., GWAS). Mutations in multiple genes and loci were reported in 87 and 12 studies, respectively. A significant association of *IRF6*, *MSX1* and *NECTIN1* with CL/P has been reported in several GWAS in different populations (Additional file 1: Table S4). However, no significant association was reported in several studies conducted in Danish, Mesoamerican, and African populations for *IRF6*, American and Latvian populations for *MSX1*, and Taiwanese population for *NECTIN1* (Additional file 1: Table S6).

**Table 1:** Summary of genes associated with cleft lip with/without cleft palate in humans

Gene symbol	Gene name	Loci	Syndromic/ Nonsyndromic
ABCA4	ATP binding cassette subfamily A member 4	1p22.1	Nonsyndromic
ABCB1	ATP binding cassette subfamily B member 1	7q21.12	Nonsyndromic
ADAM3A	ADAM metallopeptidase domain 3A	8p11.22	Nonsyndromic
ACSS2	Acyl-coenzyme A synthetase short-chain family member 2	20q11.22	Nonsyndromic
ADAM5	ADAM metallopeptidase domain 5	8p11.22	Nonsyndromic
ADAMTS20	ADAM metallopeptidase with thrombospondin type 1 motif 20	12q12	Nonsyndromic
ADGRL2	Adhesion G protein-coupled receptor L2	1p31.1	Nonsyndromic
ADH1C	Alcohol dehydrogenase 1C (class I), gamma polypeptide	4q23	Nonsyndromic & Syndromic: Fetal alcohol syndrome
AQP7	Aquaporin 7	9p13.3	Nonsyndromic
ARHGAP29	Rho GTPase activating protein 29	1p22.1	Nonsyndromic
ASS1	Argininosuccinate synthase 1	9q34.11	Nonsyndromic
AXIN2	Axin 2	17q24.1	Nonsyndromic
BAG4	BCL2 associated athanogene 4	8p11.23	Nonsyndromic
BCL3	B-cell CLL/lymphoma 3	19q13.32	Nonsyndromic
BHMT2	Betaine-homocysteine S-methyltransferase 2	5q14.1	Nonsyndromic
BLM	Bloom syndrome RecQ like helicase	15q26.1	Nonsyndromic
BMP4	Bone morphogenetic protein 4	14q22.2	Nonsyndromic
BMPR1B	Bone morphogenetic protein receptor type 1B	4q22.3	Nonsyndromic
BRIP1	BRCA1 interacting protein C-terminal helicase 1	17q23.2	Nonsyndromic
CBS	Cystathionine-beta-synthase	21q22.3	Nonsyndromic
CDH1	Cadherin 1	16q22.1	Nonsyndromic
CDH2	Cadherin 2	18q12.1	Nonsyndromic
CENPJ	Centromere protein J	13q12.12-q12.13	Nonsyndromic
CHD7	Chromodomain helicase DNA binding protein 7	8q12.2	Syndromic: CHARGE syndrome
CLPTM1	CLPTM1, transmembrane protein	19q13.32	Nonsyndromic
COL4A2	Collagen type IV alpha 2 chain	13q34	Nonsyndromic
COL4A3	Collagen type IV alpha 3 chain	2q36.3	Nonsyndromic
COL4A4	Collagen type IV alpha 4 chain	2q36.3	Nonsyndromic
COL21A1	Collagen type XXI alpha 1 chain	6p12.1; 6p12.3-p11.2	Nonsyndromic
CR1	Complement C3b/C4b receptor 1 (Knops blood group)	1q32.2	Syndromic: Van der Woude syndrome
CRISPLD2	Cysteine rich secretory protein LCCL domain containing 2	16q24.1	Nonsyndromic
CYP1A1	Cytochrome P450 family 1 subfamily A member 1	15q24.1	Nonsyndromic
CYP2E1	Cytochrome P450 family 2 subfamily E member 1	10q26.3	Nonsyndromic
DCAF7	DDB1 and CUL4 associated factor 7	17q23.3	Nonsyndromic
DHFR	Dihydrofolate reductase	5q14.1	Nonsyndromic
DICER1	Dicer 1, ribonuclease III	14q32.13	Nonsyndromic
DLX1	Distal-less homeobox 1	2q31.1	Nonsyndromic
DMD	Dystrophin	Xp21.2-p21.1	Nonsyndromic
DVL2	Dishevelled segment polarity protein 2	17p13.1	Nonsyndromic
E2F1	E2F transcription factor 1	20q11.22	Nonsyndromic
EFNB1	Ephrin B1	Xq13.1	Syndromic: Craniofrontonasal syndrome
EIF2B3	Eukaryotic translation initiation factor 2B subunit gamma	1p34.1	Nonsyndromic
EGF61	Epidermal growth factor 61	4q25	Nonsyndromic
ESRRG	Estrogen related receptor gamma	1q41	Nonsyndromic
EVC	EvC ciliary complex subunit 1	4p16.2	Nonsyndromic
EVC2	EvC ciliary complex subunit 2	4p16.2	Nonsyndromic
EYA1	EYA transcriptional coactivator and phosphatase 1	8q13.3	Nonsyndromic
F13A1	Coagulation factor XIII A chain	6p25.1	Nonsyndromic
FAM49A	Family with sequence similarity 49 member A	2p24.2	Nonsyndromic
FGF1	Fibroblast growth factor 1	5q31.3	Nonsyndromic
FGF2	Fibroblast growth factor 2	4q28.1	Nonsyndromic
FGF3	Fibroblast growth factor 3	11q13.3	Nonsyndromic
FGF10	Fibroblast growth factor 10	5p12	Nonsyndromic
FGF18	Fibroblast growth factor 18	5q35.1	Nonsyndromic
FGFR1	Fibroblast growth factor receptor 1	8p11.23	Nonsyndromic & Syndromic: Kallmann syndrome
FGFR2	Fibroblast growth factor receptor 2	10q26.13	Nonsyndromic
FOXE1	Forkhead box E1	9q22.33	Nonsyndromic
FOXF2	Forkhead box F2	6p25.3	Nonsyndromic
FOXG1	Forkhead box G1	14q12	Nonsyndromic
FOXP2	Forkhead box protein P2	7q31.1	Nonsyndromic
FZD6	Frizzled class receptor 6	8q22.3	Nonsyndromic
GABRB3	Gamma-aminobutyric acid type A receptor beta 3 subunit	15q12	Nonsyndromic
GAD1	Glutamate decarboxylase 1	2q31.1	Nonsyndromic
GCH1	GTP cyclohydrolase 1	14q22.2	Nonsyndromic
GLI2	GLI family zinc finger 2	2q14.2	Nonsyndromic
GREM1	Gremlin 1, DNA family BMP antagonist	15q13.3	Nonsyndromic
GSTM1	Glutathione S-transferase mu 1	1p13.3	Nonsyndromic
GSTP1	Glutathione S-transferase pi 1	11q13.2	Nonsyndromic
GSTT1	Glutathione S-transferase theta 1	22q11.23	Nonsyndromic
HECTD1	HECT domain E3 Ubiquitin protein ligase 1	14q12	Nonsyndromic
HKDC1	Hexokinase domain containing 1	10q22.1	Nonsyndromic
IRF6	Interferon regulatory factor 6	1q32.2	Nonsyndromic
JAG2	Jagged2	14q32.33	Nonsyndromic
JARID2	Jummonji and AT-rich interaction domain containing 2	6p22.3	Nonsyndromic
KIF2A	Kinesin family member 2A	5q12.1	Nonsyndromic
KIF7	Kinesin family member 7	15q26.1	Nonsyndromic
KISS1R	KISS1 receptor	19p13.3	Syndromic: Kallmann syndrome
KRT18	Keratin 18	12q13.13	Nonsyndromic
LACTB	Lactamase beta	15q22.2	Nonsyndromic
LHX8	LIM homeobox 8	1p31.1	Nonsyndromic
LPHN2	Adhesion G protein-coupled receptor L2	1p31.1	Nonsyndromic
MAFB	MAF bZIP transcription factor B	20q12	Nonsyndromic
MGAM	Maltase-glucoamylase	7q34	Nonsyndromic
MID1	Midline 1	Xp22.2	Nonsyndromic & Syndromic: Opitz G/BBB syndrome
MMP3	Matrix metallopeptidase 3	11q22.2	Nonsyndromic
MMP9	Matrix metallopeptidase 9	20q13.12	Nonsyndromic
MROH7	Maestro heat like repeat family member 7	1p32.3	Nonsyndromic
MRPL53	Mitochondrial ribosomal protein L53	2p13.1	Nonsyndromic
MSX1	msh homeobox 1	4p16.2	Nonsyndromic
MSX2	msh homeobox 2	5q35.2	Nonsyndromic
MTR	5-methyltetrahydrofolate-homocysteine methyltransferase	1q43	Nonsyndromic
MTRR	5-methyltetrahydrofolate-homocysteine methyltransferase reductase	5p15.31	Nonsyndromic
MYH9	Myosin heavy chain 9	22q12.3	Nonsyndromic
MTHFD1	Methylenetetrahydrofolate dehydrogenase, cyclohydrolase and formyltetrahydrofolate synthetase 1	14q23.3	Nonsyndromic
MTHFR	Methylenetetrahydrofolate reductase	1p36.22	Nonsyndromic
NAT1	N-acetyltransferase 1	8p22	Nonsyndromic
NAT2	N-acetyltransferase 2	8p22	Nonsyndromic
NECTIN1	Nectin cell adhesion molecule 1	11q23.3	Nonsyndromic
NECTIN2	Nectin cell adhesion molecule 2	19q13.32	Nonsyndromic
NECTIN3	Nectin cell adhesion molecule 3	3q13.13	Nonsyndromic
NOG	Noggin	17q22	Nonsyndromic
NTN1	Netrin 1	17p13.1	Nonsyndromic
OFC1	Orofacial cleft 1	6p24	Nonsyndromic
OFC2	Orofacial cleft 2	2p14-p13	Nonsyndromic
PAH	Phenylalanine hydroxylase	12q23.2	Nonsyndromic
PARVA	Parvin alpha	11p15.3	Nonsyndromic
PTCH1	Patched 1	9q22.32	Nonsyndromic
PAX3	Paired box 3	2q36.1	Nonsyndromic
PAX7	Paired box 7	1p36.13	Nonsyndromic
PAX9	Paired box 9	14q13.3	Nonsyndromic
PCYT1A	Phosphate cytidylyltransferase 1, choline, alpha	3q29	Nonsyndromic
PDGFC	Platelet derived growth factor C	4q32.1	Nonsyndromic
PEMT	Phosphatidylethanolamine N-methyltransferase	17p11.2	Nonsyndromic
PKP1	Plakophilin 1	1q32.1	Nonsyndromic
PHF8	PHD finger protein 8	Xp11.22	Nonsyndromic
PHYH	Phytanoyl-CoA 2-hydroxylase	10p13	Nonsyndromic
RPS26	Ribosomal Protein S26	12q13.2	Nonsyndromic
PRSS1	Protease, serine 1	7q34	Nonsyndromic
PRSS35	Protease, serine 35	6q14.2	Nonsyndromic
RAD51	RAD51 recombinase	15q15.1	Nonsyndromic
RAD54B	RAD54 homolog B	8q22.1	Nonsyndromic
RARA	Retinoic Acid Receptor Alpha	17q21.2	Nonsyndromic
RECQL5	RecQ like helicase 5	17q25.1	Nonsyndromic
REG3A	Regenerating family member 3 alpha	2p12	Nonsyndromic
REN	Renin	1q32.1	Syndromic: Van der Woude syndrome
RFC1	Replication factor C subunit 1	4p14	Nonsyndromic
RHPN2	Rhophilin Rho GTPase binding protein 2	19q13.11	Nonsyndromic
RUNX2	Runt related transcription factor 2	6p21.1	Nonsyndromic
RYK	Receptor-like tyrosine kinase	3q22.2	Nonsyndromic
SATB2	SATB homeobox 2	2q33.1	Nonsyndromic
SEC16A	SEC 16 homolog A, endoplasmic reticulum export factor	9q34.3	Nonsyndromic
SERPINA6	Serpin family A member 6	14q32.13	Nonsyndromic
SLC6A4	Solute carrier family 6 member 4	17q11.2	Nonsyndromic
SMAD1	SMAD family member 1	4q31.21	Nonsyndromic
SMAD2	SMAD family member 2	18q21.1	Nonsyndromic
SOX9	SRY-box 9	17q24.3	Syndromic: Pierre-Robin Syndrome
SPRY1	Sprouty RTK signaling antagonist 1	4q28.1	Nonsyndromic
SUMO1	Small ubiquitin-like modifier 1	2q33.1	Nonsyndromic
STK32B	Serine/threonine kinase 32B	4p16.2	Nonsyndromic
SYNE3	Spectrin repeat containing nuclear envelop family member 3	14q32.13	Nonsyndromic
TAF1B	TATA-box binding protein associated factor, RNA polymerase I subunit B	2p25.1	Nonsyndromic
TANC2	Tetratricopeptide repeat, ankyrin repeat and coiled-coil containing 2	17q23.2-q23.3	Nonsyndromic
TBX22	T-box 22	Xq21.1	Nonsyndromic
TCN2	Transcobalamin 2	22q12.2	Nonsyndromic
TEX11	Testis expressed 11	Xq13.1	Nonsyndromic
TFAP2A	Transcription factor AP-2 alpha	6p24.3	Nonsyndromic
TGFA	Transforming growth factor alpha	2p13.3	Nonsyndromic
TGFB1	Transforming growth factor beta 1	2p13.3	Nonsyndromic
TGFB2	Transforming growth factor beta 2	1q41	Nonsyndromic
TGFB3	Transforming growth factor beta 3	14q24.3	Nonsyndromic
TIMP2	TIMP metallopeptidase inhibitor 2	17q25.3	Nonsyndromic
TMEM19	transmembrane protein 19	12q21.1	Nonsyndromic
TNS1	Tensin 1	2q35	Nonsyndromic
TOX3	TOX high mobility group box family member 3	16q12.1	Nonsyndromic
TP63	Tumor protein p63	3q28	Syndromic: Hay wells or AEC syndrome
TPH2	Tryptophan hydroxylase 2	12q21.1	Nonsyndromic
TPM1	Tropomyosin 1 (alpha)	15q22.2	Nonsyndromic
TULP4	Tubby like protein 4	6q25.3	Nonsyndromic
TYMS	Thymidylate synthase	18p11.32	Nonsyndromic
VAX1	Ventral anterior homeobox 1	10q25.3	Nonsyndromic
VAX2	Ventral anterior homeobox 2	2p13.3	Nonsyndromic
VWA8	Von Willebrand factor A domain-containing protein 8	13q14.11	Nonsyndromic
WNT3	Wnt family member 3	17q21.31-q21.32	Nonsyndromic
WNT3A	Wnt family member 3A	1q42.13	Nonsyndromic
WNT5A	Wnt family member 5A	3p14.3	Nonsyndromic
WNT5B	Wnt family member 5B	12p13.33	Nonsyndromic
WNT6	Wnt family member 6	2q35	Nonsyndromic
WNT9B	Wnt family member 9B	17q21.32	Nonsyndromic
WNT10A	Wnt family member 10A	2q35	Nonsyndromic
WNT11	Wnt family member 11	11q13.5	Nonsyndromic
YOD1	TOD1 deubiquitinase	1q32.1	Nonsyndromic
ZNF385B	Zinc finger protein 385B	2q31.2-q31.3	Nonsyndromic

Due to space limits, the details (e.g. publication ID) of each gene were not provided in this table

### GO term enrichment analysis

We analyzed CL/P genes enriched in the GO terms to assess the functional features of CL/P genes (Additional file 1: Tables S9–S12). The most specific enriched terms among GO biological processes showed strong association with development and morphogenesis of other organs (Additional file 1: Tables S9 and S10). These results suggest that CL/P genes may potentially cause additional developmental disorders while 70% of CL/P cases are non-syndromic without any additional birth defects. In non-syndromic CL/P cases, molecules that are specifically expressed in the lip region during lip formation may help explain the molecular mechanism in regard to how only lip formation is affected. Lip formation involves the growth and fusion of maxillary and nasal processes during embryogenesis [25]. We identified a strong association with positive and negative regulators of several cellular processes, including cell proliferation, apoptosis, differentiation, and epithelial-to-mesenchymal transition. We also found that genes involved in folic acid metabolic process were significantly enriched (7 CL/P genes) (Additional file 1: Table S10). The current approach for the prevention of neural tube defects and CL/P is folic acid supplementation [26]. Our results suggest that some individuals with CL/P have defects in folic acid metabolism, and that a folic acid supplement can potentially prevent CL/P.

Among the enriched GO terms in its domain Molecular Function (MF) (Additional file 1: Tables S9 and S11), we observed enrichment of several terms related to molecular binding: transcription factor activity, sequence-specific binding (28 CL/P genes), sequence-specific DNA binding (23 CL/P genes), chromatin binding (16 CL/P genes), and binding to Frizzled, a family of G protein-coupled receptors for WNT ligands (11 CL/P genes). The remaining enriched terms in the MF domain included: protein homodimerization activity (26 CL/P genes); growth factor activity (13 CL/P genes), which is induced by FGF, BMP, TGFβ and PDGF; protein tyrosine kinase activity (10 CL/P genes), 1- phosphatidylinositol-3 kinase activity (8 CL/P genes), and phosphatidylinositol-4,5-bisphosphate 3-kinase activity (8 CL/P genes) induced by FGF signaling; and others. Thus, the GO MF term analysis highlighted the contribution of morphologic factors involved in WNT, FGF, BMP, TGFβ, and PDGF signaling pathways.

Among GO terms representing cellular components (Additional file 1: Tables S9 and S12), most terms were enriched in extracellular components: extracellular region (48 CL/P genes), extracellular space (36 CL/P genes), cell surface (27 CL/P genes), and proteinaceous extracellular matrix (ECM) (22 CL/P genes). In GO terms, many growth factors including WNT, FGF, BMP, TGFβ, and PDGF and their extracellular regulators (e.g. ECM) were identified in these groups. These findings are in agreement with the fact that growth factors are activated in the extracellular space.

### Human phenotype ontology enrichment analysis

We used the HPO database to investigate which human phenotype terms were enriched for the identified set of CL/P candidate genes. The most enriched phenotypic term was abnormal number of teeth (28 genes), followed by CL (23 genes), cleft upper lip (22 genes), reduced number of teeth (25 genes), and oral cleft (36 genes) (Additional file 1: Table S13). Among the top 30 results, most phenotypes were directly related to CL/P, congenital dental disorders, or congenital abnormalities of the fingers.

### KEGG pathway analysis

We hypothesized that CL/P genes share common features among wide arrays of biological functions and pathways. We therefore examined which biological pathways were enriched with CL/P genes by using the DAVID bioinformatics tool and the canonical pathways from the KEGG database (Additional file 1: Table S14). The regulatory pathway annotation was performed based on the score and visualization of the pathways used in the KEGG database. Among the cellular functions in KEGG pathways, 19 pathways were statistically significant in the enrichment of CL/P genes (FDR < 5%). Seven of these pathways were related to cancer: pathways in cancer (43 CL/P genes), basal cell carcinoma (16 CL/P genes), proteoglycans in cancer (16 CL/P genes), melanoma (10 CL/P genes), pancreatic cancer (8 CL/P genes), bladder cancer (6 CL/P genes), and colorectal cancer (7 CL/P genes). Previous population-based studies suggest that individuals with CL/P have a higher risk of cancer in the breast, brain, and lung later in life [27–29]. Among them, molecules related to Hippo, FGF, WNT, and TGFβ signaling are highlighted in these cancer-related pathways. These cellular signaling pathways were significantly involved in CL/P: Hippo signaling pathway (22 CL/P genes), signaling pathways regulating pluripotency of stem cells (21 CL/P genes), phosphoinositide 3-kinase (PI3K)- Akt signaling pathway (15 CL/P genes), mitogen-activated protein kinase (MAPK) signaling pathway (13 CL/P genes), WNT signaling pathway (11 CL/P genes), and TGF-β signaling pathway (9 CL/P genes).

### Genotype-phenotype association analysis

To determine the contribution of genetic variations to CL/P, a genotype-phenotype association analysis was conducted using GWAS for the genetics of orofacial clefts and related phenotypes (e.g. CL/P) available from dbGaP (dbGaP accession phs000774.v1.p1). By PLINK analysis, we identified 2975 cases vs 8751 controls out of 11,925 individuals. We investigated whether single nucleotide polymorphisms (SNPs) mapped to the CL/P-candidate genes are associated with human CL/P phenotypes using TDT of The Genetics of Orofacial Clefts and Related Phenotypes GWAS dataset. This method is appropriate for statistical imbalances between transmitted and non-transmitted alleles in parent-child trios. We investigated all directly genotyped (i.e. not imputed) variants within the genes found in the systematic review. A total of 5437 variants from 179 genes were included in the association analysis, while some genes did not have the genotyped SNPs in this dataset and some SNPs did not present any variations in the analyzed samples. Because most SNPs in the same gene have strong linkage disequilibrium, we set our candidate-wise significance threshold at 2.79 × 10^− 4^ by using the Bonferroni level with the number of genes tested (0.05/179). We identified 56 SNPs from 12 genes with *p*-values smaller than 2.79 × 10^− 4^ (Table 2). The top association signals were from *IRF6* and *NTN1* (Netrin 1), which also showed nominal genome-wide significance in the GWAS dataset (*p* < 5 × 10^− 8^). We also observed gene-wide significant signals (*p* < 2.5 × 10^− 6^) from *ABCA4* (ATP-binding cassette, sub-family A, member 4) and *PAX7* (paired box 7). Although *ADAM3A* (ADAM metallopeptidase domain 3A), *FOXE1* (forkhead box E1), *MSX2* (msh homeobox 2), *MTHFR* (methylenetetrahydrofolate reductase), *TP63* (tumor protein p63), *TPM1* (tropomyosin 1), *VAX1* (ventral anterior homeobox 1), and *WNT9B* (wingless-type MMTV integration site family, member 9B) did not reach the genome-wide nor gene-wide significance thresholds, these genes reached candidate-wise significance level (*p* < 2.79 × 10^− 4^).

**Table 2:** Genotype-phenotype association

Top marker	Type	Gene	*p*-value
rs1044516	UTR3	*IRF6*	2.66 × 10^−9^
rs1880646	Intron	*NTN1*	3.02 × 10^−9^
rs4147827	Intron	*ABCA4*	8.51 × 10^−7^
rs742071	Intron	*PAX7*	2.09 × 10^−6^
rs6474148	Intron	*ADAM3A*	3.26 × 10^−6^
rs1443435	UTR3	*FOXE1*	1.39 × 10^−5^
rs6585429	Intron	*VAX1*	2.28 × 10^−5^
rs2066462	Silent	*MTHFR*	7.38 × 10^−5^
rs3803499	Intron	*TPM1*	1.91 × 10^−4^
rs1515497	Intron	*TP63*	2.19 × 10^−4^
rs14459	UTR3	*MSX2*	2.34 × 10^−4^
rs4968282	Intron	*WNT9B*	2.65 × 10^−4^

### Environmental and epigenetic factors

In addition to gene mutations, genetic background such as ethnicity, population and gender have substantial influence on the birth prevalence of CL/P. Environmental factors such as maternal age, smoking, alcohol consumption, obesity, and micronutrient deficiencies are known to be strong susceptibility risk factors. Recent studies suggest that environmental factors can interact with the epigenetic system and control gene expression at the post- transcriptional level [30–32]. To explore the degree to which miRNAs regulate the expression of CL/P genes, we performed a bioinformatics analysis to identify miRNAs whose target genes are statistically enriched with CL/P genes (Table 3). By applying the adjusted *p*-value (FDR) < 0.1, a total of 16 miRNAs were significantly enriched with the targeted CL/P genes. These 16 miRNAs were categorized into 10 known and one unknown miRNA families: the miR-27 family (hsa-miR- 27b- 3p, 23 CL/P genes), miR-124 family (hsa- miR- 124-3p, 29 CL/P genes), miR-154 family (hsa-miR-369-3p, 13 CL/P genes; hsa- miR-655-3p, 14 CL/P genes; hsa-miR-300, 18 CL/P genes; hsa-miR-381-3p, 18 CL/P genes), miR-203 family (hsa-miR-203a-3p, 9 CL/P genes), miR-368 family (hsa-miR-376b-3p, 10 CL/P genes), miR-374 family (hsa-miR- 374a-5p, 22 CL/P genes; hsa-miR-374b-5p, 22 CL/P genes), miR-497 family (hsa-miR-497-5p, 25 CL/P genes), miR-503 family (hsa-miR-503-5p, 10 CL/P genes), miR-550 family (hsa-miR-550a-5p, 5 CL/P genes; hsa-miR-550a-3-5p, 5 CL/P genes), miR-1271 family (hsa-miR-1271-3p, 5 CL/P genes), and an unknown family (hsa-miR-3678-3p, 5 CL/P genes). Notably, except for hsa-miR-27b-3p [33], these miRNAs have not yet been reported in CL/P. In the enrichment analysis, CL/P genes regulated by multiple miRNAs were: *EN2* (targeted by 6 miRNA families), *FZD6* (targeted by 5 miRNA families), *HECTD1* (targeted by 5 miRNA families), and *YOD1* (targeted by 5 miRNA families) (Table 4). Because gene expression of *EN2*, *FZD6*, *HECTD1*, and *YOD1* is regulated by several miRNAs, expression of these genes may be more susceptible to environmental factors during lip formation.

**Table 3:** MicroRNA (miRNA) enrichment analysis of 161 human CL/P genes (FDR < 0.1)

miRNA family ID	miRNA name	Gene symbol	FDR (BH)
miR-154	hsa-miR-369-3p	*CHD7*, *DMD*, *EYA1*, *GABRB3*, *GREM1*, *HOXB3*, *NOG*, *PAX6*, *TIMP2*, *TULP4*, *BAG4*, *FZD6*, *YOD1*	0.03
miR-154	hsa-miR-655-3p	*BCL2*, *DMD*, *EN2*, *GREM1*, *HOXB3*, *MAFB*, *MID1*, *NTN1*, *PAX6*, *SATB2*, *TULP4*, *CYP1A1*, *FZD6*, *YOD1*	0.03
miR-374	hsa-miR-374a-5p	*CHD7*, *DMD*, *EYA1*, *FGFR2*, *FOXG1*, *HECTD1*, *HOXB3*, *JARID2*, *MSX1*, *NOG*, *NTN1*, *PAX6*, *RAD51*, *RHPN2*, *RUNX2*, *TGFA*, *TNS1*, *WNT5A*, *WNT5B*, *YOD1*, *EN2*, *FZD6*	0.07
miR-374	hsa-miR-374b-5p	*CHD7*, *DMD*, *EYA1*, *FGFR2*, *FOXG1*, *HECTD1*, *HOXB3*, *JARID2*, *MSX1*, *NOG*, *NTN1*, *PAX6*, *RAD51*, *RHPN2*, *RUNX2*, *TGFA*, *TNS1*, *WNT5A*, *WNT5B*, *YOD1*, *EN2*, *FZD6*	0.07
miR-497	hsa-miR-497-5p	*AXIN2*, *BAG4*, *BCL2*, *CHD7*, *CRISPLD2*, *EN2*, *EYA1*, *FGF1*, *FGF2*, *FGFR1*, *FGFR2*, *FOXP2*, *FZD6*, *HECTD1*, *JARID2*, *PAX7*, *RHPN2*, *SATB2*, *SLC6A4*, *WNT3A*, *YOD1*, *MTHFR*, *RUNX2*, *TFAP2A*, *TPM1*	0.07
miR-124	hsa-miR-124-3p	*GABRB3*, *GCH1*, *JAG2*, *KIF2A*, *MTR*, *MYH9*, *ROR2*, *RYK*, *TANC2*, *ARHGAP29*, *COL4A4*, *DVL2*, *EFNB1*, *FGF1*, *FGFR1*, *FOXF2*, *GREM1*, *HOXB3*, *KIF7*, *PDGFC*, *PEMT*, *PKP1*, *RAD51*, *RUNX2*, *SOX9*, *TBX22*, *TP63*, *TPM1*, *WNT5B*	0.09
miR-1271	hsa-miR-1271-3p	*CBS*, *DCAF7*, *FZD6*, *RAD51*, *YOD1*	0.09
miR-203	hsa-miR-203a-3p	*E2F1*, *EN2*, *GREM1*, *HECTD1*, *KIF2A*, *PAX6*, *RUNX2*, *SMAD2*, *TP63*	0.09
miR-27	hsa-miR-27b-3p	*BCL3*, *CHD7*, *COL21A1*, *DVL2*, *EN2*, *EYA1*, *FGF1*, *FOXP2*, *GABRB3*, *GCH1*, *GREM1*, *HOXB3*, *PAX9*, *RARA*, *SATB2*, *SMAD1*, *STK32B*, *SMAD2*, *DCAF7*, *PAX3*, *PAX7*, *SEC16A*, *WNT9B*	0.09
miR-154	hsa-miR-300	*CR1*, *DVL2*, *FGF1*, *FGFR2*, *FOXF2*, *FOXP2*, *GABRB3*, *GAD1*, *HECTD1*, *JAG2*, *MID1*, *PDGFC*, *TANC2*, *TGFB3*, *WNT5A*, *CRISPLD2*, *GREM1*, *PHF8*	0.09
NA	hsa-miR-3678-3p	*E2F1*, *GABRB3*, *JARID2*, *SLC6A4*, *YOD1*	0.09
miR-368	hsa-miR-376b-3p	*CRISPLD2*, *EN2*, *FGFR1*, *MYH9*, *PAX3*, *PAX6*, *PRSS35*, *TANC2*, *FGF2*, *FOXG1*	0.09
miR-154	hsa-miR-381-3p	*CR1*, *DVL2*, *FGF1*, *FGFR2*, *FOXF2*, *FOXP2*, *GABRB3*, *GAD1*, *HECTD1*, *JAG2*, *MID1*, *PDGFC*, *TANC2*, *TGFB3*, *WNT5A*, *CRISPLD2*, *GREM1*, *PHF8*	0.09
miR-503	hsa-miR-503-5p	*HECTD1*, *JARID2*, *MAFB*, *WNT3A*, *BCL2*, *DHFR*, *FGF2*, *FGFR1*, *SMAD2*, *TFAP2A*	0.09
miR-550	hsa-miR-550a-3-5p	*CBS*, *DCAF7*, *FZD6*, *RAD51*, *YOD1*	0.09
miR-550	hsa-miR-550a-5p	*CBS*, *DCAF7*, *FZD6*, *RAD51*, *YOD1*	0.09

*BH* Benjamini–Hochberg adjustment for multiple test correction [23]

**Table 4:** Human CL/P genes targeted by at least two microRNA (miRNA) families

Gene	# miRNA families	miRNA family
*EN2*	6	miR-154, miR-203, miR-27, miR-368, miR-374, miR-497
*YOD1*	5	miR-1271, miR-154, miR-374, miR-497, miR-550
*FZD6*	5	miR-1271, miR-154, miR-374, miR-497, miR-550
*HECTD1*	5	miR-154, miR-203, miR-374, miR-497, miR-503
*GREM1*	4	miR-124, miR-154, miR-203, miR-27
*HOXB3*	4	miR-124, miR-154, miR-27, miR-374
*PAX6*	4	miR-154, miR-203, miR-368, miR-374
*RAD51*	4	miR-124, miR-1271, miR-374, miR-550
*CHD7*	4	miR-154, miR-27, miR-374, miR-497
*EYA1*	4	miR-154, miR-27, miR-374, miR-497
*RUNX2*	4	miR-124, miR-203, miR-374, miR-497
*FGF1*	4	miR-124, miR-154, miR-27, miR-497
*FGFR1*	4	miR-124, miR-368, miR-497, miR-503
*GABRB3*	3	miR-124, miR-154, miR-27
*FGFR2*	3	miR-154, miR-374, miR-497
*JARID2*	3	miR-374, miR-497, miR-503
*CRISPLD2*	3	miR-154, miR-368, miR-497
*FOXP2*	3	miR-154, miR-27, miR-497
*TANC2*	3	miR-124, miR-154, miR-368
*DVL2*	3	miR-124, miR-154, miR-27
*DCAF7*	3	miR-1271, miR-27, miR-550
*BCL2*	3	miR-154, miR-497, miR-503
*SATB2*	3	miR-154, miR-27, miR-497
*FGF2*	3	miR-368, miR-497, miR-503
*SMAD2*	3	miR-203, miR-27, miR-503
*DMD*	2	miR-154, miR-374
*WNT5A*	2	miR-154, miR-374
*NOG*	2	miR-154, miR-374
*NTN1*	2	miR-154, miR-374
*FOXG1*	2	miR-368, miR-374
*RHPN2*	2	miR-374, miR-497
*WNT5B*	2	miR-124, miR-374
*JAG2*	2	miR-124, miR-154
*FOXF2*	2	miR-124, miR-154
*PDGFC*	2	miR-124, miR-154
*CBS*	2	miR-1271, miR-550
*BAG4*	2	miR-154, miR-497
*MAFB*	2	miR-154, miR-503
*PAX7*	2	miR-27, miR-497
*WNT3A*	2	miR-497, miR-503
*TFAP2A*	2	miR-497, miR-503
*TPM1*	2	miR-124, miR-497
*GCH1*	2	miR-124, miR-27
*KIF2A*	2	miR-124, miR-203
*MYH9*	2	miR-124, miR-368
*TP63*	2	miR-124, miR-203
*PAX3*	2	miR-27, miR-368

### Experimental validation

**Fig. 2:**
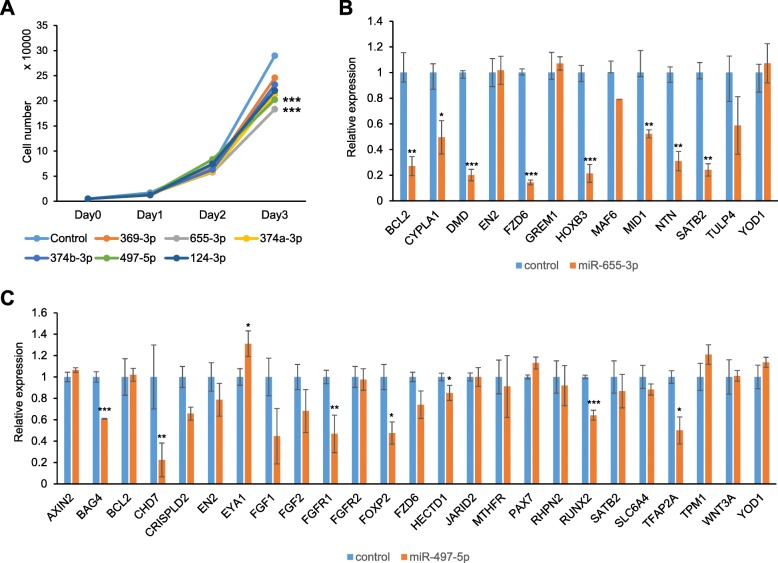
Experimental validation of predicted miRNAs. **a** Cell proliferation assays in human lip fibroblasts treated with the indicated mimics of miRNAs. Negative control (control, light blue), miR-369-3p (orange), miR-655-3p (gray), miR-374a-3p (yellow), miR-374b-3p (blue), miR-497-5p (light green), and miR-124-3p (dark blue). *** *p* < 0.001. **b** Quantitative RT-PCR for the indicated genes after treatment with negative control (light blue) or miR-655-3p mimic (orange). * *p* < 0.05, ** *p* < 0.01, *** *p* < 0.001. **c** Quantitative RT-PCR for the indicated genes after treatment with negative control (light blue) or miR-497-5p mimic (orange). * *p* < 0.05, ** *p* < 0.01, *** *p* < 0.001

miRNA regulates expression of its genes anti- correlationally [34]. To test whether induction of potential CL/P-candidate miRNAs caused proliferation defects through the suppression of target gene expression, we treated cultured human lip fibroblasts with each miRNA mimic. The miR-497-5p and miR-655-3p mimics were most significantly inhibited cell proliferation in human lip fibroblasts (Fig. 2a). To identify target genes regulated by either miR-497-5p or miR-655-3p, we performed quantitative RT-PCR analyses for the predicted target genes (*AXIN2*, *BAG4*, *BCL2*, *CHD7*, *CRISPLD2*, *EN2*, *EYA1*, *FGF1*, *FGF2*, *FGFR1*, *FGFR2*, *FOXP2*, *FZD6*, *HECTD1*, *JARID2*, *MTHFR*, *PAX7*, *RHPN2*, *RUNX2*, *SATB2*, *SLC6A4*, *TFAP2A*, *TPM1*, *WNT3A*, and *YOD1* for miR-497-5p; and *BCL2*, *CYP1A1*, *DMD*, *EN2*, *FZD6*, *GREM1*, *HOXB3*, *MAFB*, *MID1*, *NTN1*, *PAX6*, *SATB2*, *TULP4*, and *YOD1* for miR-655-3p) in human lip fibroblasts after treatment with mimics of either miR-497-5p or miR-655-3p. The expression of almost target genes except *EN2*, *GREM1*, *MAF6*, *TULP4* and *YOD1* was significantly downregulated by treatment with miR- 655-3p mimic (Fig. 2b). The expression of target genes (*BAG4*, *CHD7*, *CRISPLD2*, *FGFR1*, *FOXP2*, *HECTD1*, *RUNX2*, and *TFAP2A*) was significantly downregulated by treatment with miR-497-5p mimic (Fig. 2c). *PAX6* was excluded because its expression is restricted in the anterior ectoderm during early embryogenesis and the ectoderm of craniofacial surface during craniofacial development [35–37]. *EYA1* expression was unexpectedly increased after treatment with miR-497-5p mimic.

## Discussion

Orofacial cleft is described in approximately 400 known human syndromes [38–40]. Several factors have been implicated in clefting by studies of mouse models [25] and genetic screening in humans. Our literature search identified 177 genes as possible causative genes of CL/P. Our follow-up bioinformatics analysis could group genes by common features of CL/P genes in function, pathway, and miRNA regulation. As expected, the contribution of most of the highlighted pathways (e.g. FGF, Hippo, TGFβ, and WNT) to the growth and developmental process has been shown in previous mouse genetic studies for craniofacial development [25]. Cellular metabolic pathways such as one-carbon metabolism are also highlighted in KEGG pathway analysis. The one-carbon metabolism, mediated by the folate cofactor, is involved in multiple physiological processes including biosynthesis of purines and thymidine, amino acid homeostasis, epigenetic maintenance, and redox defense [41]. Mice with one-carbon metabolic aberrations (*Mthfd1l*^*−/−*^ mice) are embryonic lethal by E12.5, with craniofacial deformities including midfacial cleft [41]. However, in human studies it is still controversial in the significance of genetic mutations in genes involved in one-carbon metabolism [42–45]; further studies are necessary to reach to the conclusive evidence.

Multiple processes synthesize miRNAs and then transcribe them as long primary transcripts that are cleaved by Dicer, a type 3 ribonuclease, to produce mature, functional miRNAs. In human genetic studies, the increasing number of studies show functional significance of single- nucleotide polymorphisms (SNPs) in genes related to CL/P [46–50]. These SNPs might alter the binding activity of miRNAs. For example, previous studies show that SNPs in the miRNA-binding sites of *MSX1*, *FGF2*, *FGF5* and *FGF9* are associated with the susceptibility of nonsyndromic orofacial clefts [51, 52]. A recent study shows that plasma miRNAs are differentially expressed in nonsyndromic CP and nonsyndromic CL/P [53]. The miRNAs may also systemically regulate gene expression during embryogenesis, while some miRNAs may uniquely regulate gene expression in some particular tissues with an interaction with mRNAs expressed in a tissue-specific manner. In mice, loss of *Dicer* results in absence of mature miRNAs and, therefore, the phenotype in *Dicer* knockout mice reflects how important miRNAs are for proper development. Interestingly, cranial neural crest (CNC) cell- specific *Dicer* knockout (*Dicer*^*F/F*^*;Wnt1-Cre*) mice, but not epithelial-specific *Dicer* knockout (*Dicer*^*F/F*^*;K14-Cre*) mice, exhibit severe midfacial deformities resulting from decreased cell proliferation and increased apoptosis in the developing craniofacial regions [54–57], indicating that miRNAs have crucial roles in the fate determination of CNC cells during midfacial development [54–57]. In this study, to evaluate the function of each miRNA in cell proliferation/survival, we employed human lip fibroblasts, CNC-derived cells, for our experimental validation. The functional significance of candidate miRNAs were tested in cell proliferation/survival assays in cultured human lip fibroblasts. We found that miR-369-3p, miR-655-3p, miR-374a-5p, miR-374b-5p, and miR-497-5p that were first identified in this study as candidates involved in human CL/P suppressed cell proliferation in cultured human lip fibroblasts. Top two candidate miRNAs, miR-655-3p and miR-497-5p, were further tested in the miRNA-gene regulation assay. We found that these miRNA mimics suppressed expression of genes associated with human CL/P. Thus, the miRNAs predicted were successfully validated in our experiments. Taken together, this study provides a better understanding of CL/P as well as data that will be available for the future research of genetic approaches used to characterize individual or cluster miRNAs identified in this study.

In this study, we validated that overexpression of miR-655-3p and miR-497-5p suppressed the expression of multiple genes (*BCL2*, *CYPLA1*, *DMD*, *FZD6*, *HOXB3*, *MID1*, *NTN*, and *SATB2* by miR-655-3p; and *BAG4*, *CHD7*, *FGFR1*, *FOXP2*, *HECTD1*, *RUNX2*, and *TFAP2A* by miR-497-5p) that are associated with CL/P. Previous studies indicate that miR-655-3p is downregulated in several cancers through dysregulation of ADAM10 (a disintegrin and metalloproteinase domain-containing protein 10) and the WNT/β-catenin pathway [58, 59]. By contrast, it remains unknown how miR-655-3p expression is induced.

Previous studies also show that miR-497-5p is downregulated in several cancers [60–62]. Interestingly, miR-497-5p is upregulated during myofibroblast differentiation of lung resident mesenchymal stem cells and in the lung tissues of a pulmonary fibrosis mouse model [63]. While it remains unclear what factors and triggers induce miR-655-3p expression, increased miR-655-3p levels may result in accelerated tissue differentiation with less proliferation in various tissues. Several environmental risk factors for CL/P such as smoking, alcohol consumption and toxins [64] may cause CL/P through the upregulation of these CL/P-associated miRNAs. In addition, when the levels of these microRNAs are increased too much, multiple CL/P genes would be suppressed, as we demonstrated in this study. Our results may partially explain why individuals with CL/P show suppression of multiple CL/P genes without genetic mutations in the coding regions, which contributes to the complexity of the CL/P etiology.

In this study, we found that miR-497-5p suppressed *CHD7* expression at maximum degree of 80% in cultured human lip fibroblasts. Mutations in *CHD7* cause CHARGE syndrome, which is characterized by CL/P (in 20–36% of the cases), abnormal middle and external ear, coloboma, choanal atresia and hypoplastic semi-circular canals, rhombencephalic dysfunction, hypothalamo- hypophyseal dysfunction, mental retardation, and tracheoesophageal fistula, but do not contribute to nonsyndromic CL/P [65]. miR- 497-5p also suppressed *RUNX2* expression; SNPs in *RUNX2* are known to increase the risk of nonsyndromic CL/P. Although *FOXP2* and *BAG4* mutations are associated with nonsyndromic CL/P [66, 67], less is known about the role of these genes in CL/P development. Mouse genetic mutant models are useful tools to investigate the role of genes in lip formation, but the loss of these CL/P genes fails to cause CL/P in mice, although it has not been examined whether suppression of multiple CL/P-associated genes causes CL/P. However, mice deficient for *Tfap2a*, which is suppressed by miR-497-5p, exhibit midfacial cleft [68], suggesting that *TFAP2A* may be a principal downstream target of miR-497-5p in human lip fibroblasts. Mutations in *TFAP2A* cause Branchio-Oculo-Facial Syndrome (BOFS), which is characterized by CL/P, branchial arch defects, and ocular anomalies [69], and are associated with nonsyndromic CL/P in several populations. In addition, *Tfap2a* deficiency suppresses *Fgf8* expression in craniofacial regions in mice [68], suggesting that FGF signaling is a downstream pathway of TFAP2A. In this study, we found that miR-497-5p suppressed *FGFR1* gene expression in cultured human lip fibroblasts. Therefore, a combined downregulation of *TFAP2A* and *FGFR1* may compromise this cascade. *FGFR1* mutations are also associated with increased risk of nonsyndromic CL/P, as well as of Kallmann syndrome with CL/P [70] and Hartsfield syndrome, which is characterized by holoprosencephaly, ectrodactyly, and CL/P [71].

miR-655-3p also suppressed the expression of *FZD6*, a WNT receptor, with a maximum reduction of 85%, and of *SATB2* in cultured human lip fibroblasts. Mutations in *FZD6* are found in nonsyndromic CL/P individuals, and SNPs in *SATB2* are associated with nonsyndromic CL/P in several populations. Importantly, mice deficient for *Satb2* also show CL and CP, as seen in individuals with mutations in *SATB2*. Interestingly, wild-type mouse embryos maternally exposed to phenytoin, a drug used to prevent and control seizures but presenting congenital anomalies as common side effects, show decreased *Satb2* expression in craniofacial tissues [72]. This suggests that miR-655-3p may be upregulated in this condition of CL/P. miR-655-3p also suppressed expression of *DMD* and *MID1* in cultured human fibroblasts. These genes are located on the X chromosome so that their mutations are associated with X-linked nonsyndromic CL/P in men and women. Mutations in *MID1* are associated with the X-linked Opitz G/BBB syndrome, characterized by midline defects such as CL and CP, hypertelorism, and laryngo-trachea-esophageal (LTE) abnormalities [73, 74]. CYP1A1 is involved in drug/agent metabolism and several of these metabolites are known to be carcinogens [75]; *CYP1A1* mutations may be associated with CL/P induced with smoking and detoxification [76]. The role of other downstream targets (*NTN1* and *BCL2*) of miR-655-3p in the CL/P etiology remains largely unknown, and mice deficient for either *Ntn1* or *Bcl2* fail to display CL/P and CP.

Our systematic data collection timely summarizes the CL/P-candidate genes; however, it has some limitations. For example, some genes are from syndromes that include CL/P as a feature, but often they do not. These causative genes may be more broadly related to development, but do not contribute to CL/P; CL/P may be secondary to other defects in these syndromes. The current genetic signature of CL/P or other complex genetic diseases may be due to the bias of the type of genes that have been studied. Unbiased genome sequencing approaches will likely overcome the limitations and help us discover genes associated with CL/P. Molecular profiling of CL/P at the genomic, transcriptomic, regulatory (e.g. enhancer), epigenomic, and proteomic levels in model organisms like mice will provide us with a more detailed and accurate view of how genetic changes cause CL/P.

## Conclusion

Our bioinformatics analysis results suggest that a disruption of extracellular cues and their signaling pathways might be a major cause of CL/P, and that miRNAs might play important roles in the CL/P etiology through the regulation of CL/P genes. In this study, we found that several miRNAs suppressed cell proliferation in cultured human lip fibroblasts. Among them, we confirmed that miR-655-3p and miR497-5p negatively regulated CL/P-candidate genes in the cultured cells. This study will have potential relevance to the miRNA-gene pathways and networks, not only in CL/P but also in other organogenesis processes. Therefore, this study will contribute to a better understanding of the mechanisms of CL/P and to future clinical interventions to prevent and diagnose CL/P.

## Additional files

Additional file 1: **Table S1.** PCR primer sets used in this study. **Table S2.** Summary of databases searched. **Table S3.** Genes with significant contribution to human CL/P (identified through single gene studies). **Table S4.** Genes with significant contribution to human CL/P (identified through multiple genes studies). **Table S5.** Genes with significant contribution to human CL/P (unknown coding genes). **Table S6.** Genes without significant contribution to human CL/P. **Table S7.** CL/P candidate genes with significant signals in GWAS. **Table S8.** CL/P candidate genes without significant signals in GWAS. **Table S9.** GO terms enriched with genes associated with cleft lip with/without cleft palate (CL/P) in humans. **Table S10.** GO Biological Process terms enriched with human CL/P genes (FDR < 0.005). **Table S11.** GO Molecular Function terms enriched with human CL/P genes (FDR < 0.005). **Table S12.** GO Cellular Component terms enriched with human CL/P genes (FDR < 0.05). **Table S13.** Top 30 Human Phenotype Ontology Categories. **Table S14.** KEGG pathways enriched with genes associated with cleft lip with/without cleft palate (CL/P) in humans. (ZIP 292 kb)

## Abbreviations

CCDG: Center for Craniofacial and Dental Genetics
CL/P: Cleft lip with or without cleft palate
DAVID: Database for Annotation, Visualization and Integrated Discovery
ECM: Extracellular matrix
FDR: False Discovery Rate
GO: Gene Ontology
GWAS: Genome-wide association study
KEGG: Kyoto Encyclopedia of Genes and Genomes database
MAPK: Mitogen-activated protein kinase
MeSH: Medical Subject Headings
MF: Molecular function
miRNA: microRNA
PI3K: Phosphoinositide 3-kinase
SNP: Single nucleotide polymorphism
TDT: Transmission Disequilibrium Test
