# Supplementary material for: MicroRNA-655-3p and microRNA-497-5p inhibit cell proliferation in cultured human lip cells through the regulation of genes related to human cleft lip
Source: BMC Med Genomics. 2019 May 23;12:70. doi: 10.1186/s12920-019-0535-2 (PMC6533741; doi:10.1186/s12920-019-0535-2)
Supplement: Supplementary file 1 — Table S1. PCR primer sets used in this study. Table S2. Summary of databases searched. Table S3. Genes with significant contribution to human CL/P (identified through single gene studies). Table S4. Genes with significant contribution to human CL/P (identified through multiple genes studies). Table S5. Genes with significant contribution to human CL/P (unknown coding genes). Table S6. Genes without significant contribution to human CL/P. Table S7. CL/P candidate genes with significant signals in GWAS. Table S8. CL/P candidate genes without significant signals in GWAS. Table S9. GO terms enriched with genes associated with cleft lip with/without cleft palate (CL/P) in humans. Table S10. GO Biological Process terms enriched with human CL/P genes (FDR < 0.005). Table S11. GO Molecular Function terms enriched with human CL/P genes (FDR < 0.005). Table S12. GO Cellular Component terms enriched with human CL/P genes (FDR < 0.05). Table S13. Top 30 Human Phenotype Ontology Categories. Table S14. KEGG pathways enriched with genes associated with cleft lip with/without cleft palate (CL/P) in humans. (ZIP 292 kb) [file 12920_2019_535_MOESM1_ESM.zip › 2 Human Cleft Lip_SupplementR2.docx]

**Supplemental Information**

**MicroRNA-655-3p and microRNA-497-5p inhibit cell proliferation in cultured human lip cells through the regulation of genes related to human cleft lip**

Mona Gajera^1,#^, Neha Desai^1,#^, Akiko Suzuki^1,2^, Aimin Li^3^, Musi Zhang^1,2^, Goo Jun^4,5^, Peilin Jia^3^, Zhongming Zhao^3,4,5^, and Junichi Iwata^1,2,5,*^

**Table S1 – S14.**

**Table S1.** PCR primer sets used in this study.

**Table S2.** Summary of databases searched.

**Table S3.** Genes with significant contribution to human CL/P (identified through single gene studies).

(Excel file attached)

**Table S4.** Genes with significant contribution to human CL/P (identified through multiple genes studies).

(Excel file attached)

**Table S5.** Genes with significant contribution to human CL/P (unknown coding genes).

(Excel file attached)

**Table S6.** Genes without significant contribution to human CL/P.

(Excel file attached)

**Table S7.** CL/P candidate genes with significant signals in GWAS.

(Excel file attached)

**Table S8.** CL/P candidate genes without significant signals in GWAS.

(Excel file attached)

**Table S9.** GO terms enriched with genes associated with cleft lip with/without cleft palate (CL/P) in humans

**Table S10.** GO Biological Process terms enriched with human CL/P genes (FDR<0.005).

**Table S11.** GO Molecular Function terms enriched with human CL/P genes (FDR<0.005).

**Table S12.** GO Cellular Component terms enriched with human CL/P genes (FDR < 0.05).

**Table S13.** Top 30 Human Phenotype Ontology Categories

**Table S14.** KEGG pathways enriched with genes associated with cleft lip with/without cleft palate (CL/P) in humans.

**Table S1.** PCR primer sets used in this study

| **Gene** | **Forward primer** | **Reverse primer** |
| --- | --- | --- |
| *BCL2* | 5’-CTTTGAGTTCGGTGGGGTCA-3’ | 5’-GGGCCGTACAGTTCCACAAA-3’ |
| *DMD* | 5’-AGCTGCTGAAGTTTGTTGGTTT-3’ | 5’-CTCAATATGCTGCTTCCCAAACTTA-3’ |
| *EN2* | 5’-CTACTGTACGCGCTACTCGG-3’ | 5’-CCCGTGGCCTTCTTGATCTT-3’ |
| *GREM1* | 5’-GCCCAGCACAATGACTCAGA-3’ | 5’-TTCAGGTATTTGCGCTCCGT-3’ |
| *HOXB3* | 5’-AAATCTCCTTGGACCGGCTG-3’ | 5’-TTCCAAGCGGCTGACCTTAG-3’ |
| *MAFB* | 5’-AGAGAGAACCGAGAGGTCCC-3’ | 5’-AGCAGAGGGGAGGATCTGTT-3’ |
| *MID1* | 5’-TCATCTCCCAAGCGGAACAC-3’ | 5’-GGAGGGTTGGGAGCTGTAAG-3’ |
| *NTN1* | 5’-GGAGCCTGAAGACTGCGATT-3’ | 5’-TCACCGTGAACTTCCACCAG-3’ |
| *PAX6* | 5’-CATCACCAATCAGCATAGGAATCTG-3’ | 5’-CGCGCCCCTAGTTAAAGTCT-3’ |
| *SATB2* | 5’-GCTAGATCTGGGGACCGAAAG-3’ | 5’-GGTAACACCAAGAGCCGAGAA-3’ |
| *TULP4* | 5’-GCAGCGCCCCATCATCT-3’ | 5’-AGGCAGTGGAGAGGTAGGAG-3’ |
| *CYP1A1* | 5’-CGACACTCTTCCTTCGTCCC-3’ | 5’-TGGTTGATCTGCCACTGGTTT-3’ |
| *FZD6* | 5’-GGAGTCTTCAGCGGCTTGTAT-3’ | 5’-CTGATTGGATCTCTCTTGCGATTTC-3’ |
| *YOD1* | 5’-CGTCGCGATAACTGCAACC-3’ | 5’-GGTGATGGCGGCAATTTGG-3’ |
| *AXIN2* | 5’-TGTGAGGTCCACGGAAACTG-3’ | 5’-GGTGGGTTCTCGGGAAATGA-3’ |
| *BAG4* | 5’-GACCAGAATTGCAAGGCCAG-3’ | 5’-ATTCCAGGGTTCTGTGAAGGC-3’ |
| *CHD7* | 5’-CCCGAAGCAGGAGCTGTC-3’ | 5’-TCTGGCGCCTATTTACTGGC-3’ |
| *CRISPLD2* | 5’-GCAACTCACTGCCCAAGAATC-3’ | 5’-TTATCCACGGGCATCACGTC-3’ |
| *EYA1* | 5’-AAGAGCTGACACTGAAGCAGAG-3’ | 5’-CTCTGTTTTAACTTCGGTGCCATT-3’ |
| *FGF1* | 5’-CAGCCCTGACCGAGAAGTTT-3’ | 5’-TTTGGTGTCTGTGAGCCGTA-3’ |
| *FGF2* | 5’-GCAAAAACGGGGGCTTCTTC-3’ | 5’-TCCGTAACACATTTAGAAGCCAGTA-3’ |
| *FGFR1* | 5’-AAACCGTATGCCCGTAGCTC-3’ | 5’-GCTGCCGTACTCATTCTCCA-3’ |
| *FGFR2* | 5’-CGCTGGGGAATATACGTGCT-3’ | 5’-CTGGACTCAGCCGAAACTGT-3’ |
| *FOXP2* | 5’-GCATCTGCAACAACAGCAGG-3’ | 5’-ACAGGCACTGCAAATGTGTTG-3’ |
| *HECTD1* | 5’-ATCCCTGCAACGTCGAGC-3’ | 5’-CCGGCGTCCTCCTTTAGTTT-3’ |
| *JARID2* | 5’-TGACTCTTCTGCTCGCACTG-3’ | 5’-GAGCCAAAGACGAACGAAGC-3’ |
| *PAX7* | 5’-GTGCCCTCAGTGAGTTCGATTA-3’ | 5’-GTTCCGACTCCACATCCGAG-3’ |
| *RHPN2* | 5’-GTTCCGACTCCACATCCGAG-3’ | 5’-GCAGGGCTCATGTCGTAACT-3’ |
| *SLC6A4* | 5’-GCCCTCTGTTTCTCCTGTTCAT-3’ | 5’-CATTCAAGCGGATGTCCCCA-3’ |
| *WNT3A* | 5’-CTGGAGCTAGTGTCTCCTCTCT-3’ | 5’-CAGAGCCACGCCCTTACTG-3’ |
| *MTHFR* | 5’-GGAGGGAGGCTTCAACTACG-3’ | 5’-TGGTAGCCCTGGATGGGAAA-3’ |
| *RUNX2* | 5’-CAGAGTCAGATTACAGACCCCAG-3’ | 5’-CAGAGGTGGCAGTGTCATCA-3’ |
| *TFAP2A* | 5’-ACTCGGAGACCTCTCGATCC-3’ | 5’-GGACACGGGGCCTTTCTTAAT-3’ |
| *TPM1* | 5’-GAAGAGTTGGATCGTGCCCA-3’ | 5’-CTCGGCACATTTGCCTTCTG-3’ |
| *GAPDH* | 5’-GACAGTCAGCCGCATCTTCT-3’ | 5’-GCGCCCAATACGACCAAATC-3’ |

**Table S2.** Summary of databases searched

**a.** Ovid Medline^®^ search strategy

| 1 | Cleft Lip |
| --- | --- |
| 2 | (harelip or harelips or cheiloschisis or ((cleft or clefting) adj3 lip)).ti,ab,kw. |
| 3 | lip/ and (clefting or cleft).ti,ab,kw. |
| 4 | 1 or 2 or 3 |
| 5 | ge.fs. |
| 6 | Chromosome Deletion/ or Chromosome Mapping/ or Crosses, Genetic/ or gene amplification/ or Gene Deletion/ or Gene Expression Regulation, Developmental/ or gene expression regulation/ or gene expression/ or genetic diseases, inborn/ or genetic diseases, x-linked/ or Genetic Linkage/ or Genetic Predisposition to Disease/ or genome-wide association study/ or homeodomain proteins/ or molecular epidemiology/ or mutagenesis/ or Mutation/ or Polymorphism, Single Nucleotide/ or protein modification, translational/ or protein processing, post-translational/ or RNA processing, post-transcriptional/ or Sequence Analysis, DNA/ or Signal Transduction/ or Transcription Factors/ or transcription, genetic |
| 7 | (chromosomal or chromosome or chromosomes or gene or genes or genetic or genetics or genome or genomes or mutation or mutations or protein or proteins).ti,ab,kw. |
| 8 | microRNAs |
| 9 | (microRNA or microRNAs or miRNA or miRNAs).ti,ab,kw. |
| 10 | 5 or 6 or 7 or 8 or 9 |
| 11 | 4 and 10 |
| 12 | (11 and humans/) or (11 not animals/) |
| 13 | limit 12 to (English language) |
| 14 | 13 not (case reports or letter).pt. |

**b.** PubMed search strategy

| 1 | Cleft Lip[mesh:noexp] |
| --- | --- |
| 2 | (harelip[tiab] OR harelips[tiab] OR cheiloschisis[tiab] OR ((cleft[tiab] OR clefting[tiab]) AND lip[tiab])) |
| 3 | lip[mesh:noexp] AND (clefting[tiab] OR cleft[tiab]) |
| 4 | #1 OR #2 OR #3 |
| 5 | ge[sh] |
| 6 | Chromosome Deletion[mesh:noexp] OR Chromosome Mapping[mesh:noexp] OR Crosses, Genetic[mesh:noexp] OR gene amplification[mesh:noexp] OR Gene Deletion[mesh:noexp] OR Gene Expression Regulation, Developmental[mesh:noexp] OR gene expression regulation[mesh:noexp] OR gene expression[mesh:noexp] OR genetic diseases, inborn[mesh:noexp] OR genetic diseases, x-linked[mesh:noexp] OR Genetic Linkage[mesh:noexp] OR Genetic Predisposition to Disease[mesh:noexp] OR genome-wide association study[mesh:noexp] OR homeodomain proteins[mesh:noexp] OR molecular epidemiology[mesh:noexp] OR mutagenesis[mesh:noexp] OR Mutation[mesh:noexp] OR Polymorphism, Single Nucleotide[mesh:noexp] OR protein modification, translational[mesh:noexp] OR protein processing, post-translational[mesh:noexp] OR RNA processing, post-transcriptional[mesh:noexp] OR Sequence Analysis, DNA[mesh:noexp] OR Signal Transduction[mesh:noexp] OR Transcription Factors[mesh:noexp] OR transcription, genetic[mesh:noexp] |
| 7 | (chromosomal[tiab] OR chromosome[tiab] OR chromosomes[tiab] OR gene[tiab] OR gene[tiab]s OR genetic[tiab] OR genetics[tiab] OR genome[tiab] OR genomes[tiab] OR mutation[tiab] OR mutations[tiab] OR protein[tiab] OR proteins[tiab]) |
| 8 | MicroRNAs[mesh:noexp] |
| 9 | (microRNA[tiab] OR microRNAs[tiab] OR miRNA[tiab] OR miRNAs[tiab]) |
| 10 | #5 OR #6 OR #7 OR #8 OR #9 |
| 11 | #4 AND #10 |
| 12 | (#11 AND humans[mesh:noexp]) OR (#11 NOT animals[mesh:noexp]) |
| 13 | #12 AND English[la] |
| 14 | #13 NOT (case reports[pt] OR letter[pt]) |

**c.** Ovid EMBASE^®^ search strategy

| 1 | cleft lip |
| --- | --- |
| 2 | (harelip or harelips or cheiloschisis or (cleft adj3 lip)).ti,ab,kw. |
| 3 | congenital malformation |
| 4 | craniofacial malformation |
| 5 | (craniofacial abnormalities or craniofacial defects or craniofacial disorders or craniofacial malformations).ti,ab,kw. |
| 6 | 3 or 4 or 5 |
| 7 | lip |
| 8 | (lip or lips).ti,ab,kw. |
| 9 | 7 or 8 |
| 10 | 6 and 9 |
| 11 | 1 or 2 or 10 |
| 12 | chromosome deletion/ or chromosome deletion 13/ or chromosome deletion 22q11/ or chromosome deletion 4/ or chromosome deletion 5/ or chromosome deletion x/ or chromosome deletion y/ or interstitial chromosome deletion/ or philadelphia 1 chromosome |
| 13 | chromosome map |
| 14 | cross breeding/ or backcrossing/ or outcrossing |
| 15 | gene amplification/ or amplicon/ or ligase chain reaction/ or loop mediated isothermal amplification/ or multiplex ligation dependent probe amplification/ or multiplex polymerase chain reaction/ or nucleic acid amplification/ or nucleic acid sequence based amplification/ or reverse transcription loop mediated isothermal amplification/ or reverse transcription polymerase chain reaction/ or sequence characterized amplified region/ or telomeric repeat amplification protocol |
| 16 | gene mutation/ or allelic imbalance/ or deletion mutant/ or frameshift mutation/ or "gain of function mutation"/ or gene deletion/ or gene disruption/ or gene insertion/ or gene loss/ or indel mutation/ or "loss of function mutation"/ or missense mutation/ or mutated gene/ or nonsense mutation/ or null allele/ or point mutation/ or splicing defect |
| 17 | gene expression/ or antigen expression/ or "chromatin assembly and disassembly"/ or crispr cas system/ or enzyme induction/ or enzyme repression/ or epistasis/ or expression vector/ or gene expression regulation/ or gene overexpression/ or gene product/ or gene regulatory network/ or gene repression/ or gene silencing/ or genetic epigenesis/ or heterologous expression/ or nuclear reprogramming/ or position effect variegation/ or posttranscriptional gene silencing/ or protein induction/ or receptor down regulation/ or receptor upregulation/ or RNA interference/ or stable expression/ or transactivation/ or transient expression |
| 18 | genetic disorder/ or chromatid aberration/ or chromosome breakage/ or laminopathy |
| 19 | chromosome aberration/ or autosome aberration/ or chromosomal instability/ or mosaicism/ or nondisjunction/ or numerical chromosome aberration/ or sex chromosome aberration/ or structural chromosome aberration |
| 20 | genetic association |
| 21 | homeodomain protein/ or nucleic acid binding protein |
| 22 | molecular epidemiology |
| 23 | site directed mutagenesis/ or mutagenesis |
| 24 | mutation |
| 25 | DNA polymorphism/ or amplified fragment length polymorphism/ or restriction fragment length polymorphism/ or single nucleotide polymorphism |
| 26 | protein processing/ or protein modification |
| 27 | RNA processing/ or RNA cleavage/ or RNA editing |
| 28 | DNA sequence |
| 29 | signal transduction/ or intracellular signaling |
| 30 | transcription factor/ or genetic transcription |
| 31 | (chromosomal or chromosome or chromosomes or gene or genes or genetic or genetics or genetic or genetics or genome or genomes or mutation or mutations or protein or proteins).ti,ab,kw. |
| 32 | microRNA/ or small untranslated RNA/ or microRNA 1/ or microRNA 100/ or microRNA 101/ or microRNA 107/ or microRNA 10b/ or microRNA 122/ or microRNA 124/ or microRNA 125b/ or microRNA 126/ or microRNA 132/ or microRNA 141/ or microRNA 143/ or microRNA 145/ or microRNA 146a/ or microRNA 155/ or microRNA 15a/ or microRNA 15b/ or microRNA 16/ or microRNA182/ or microRNA 192/ or microRNA 200/ or microRNA 200a/ or microRNA 200b/ or microRNA 200c/ or microRNA 205/ or microRNA 20a/ or microRNA 20b/ or microRNA 21/ or microRNA 210/ or microRNA 214/ or microRNA 22/ or microRNA 221/ or microRNA 222/ or microRNA 223/ or microRNA 24/ or microRNA 26a/ or microRNA 29/ or microRNA 29a/ or microRNA 29b/ or microRNA 31/ or microRNA 34a/ or microRNA 375/ or microRNA 9 |
| 33 | (microRNA or microRNAs or miRNA or miRNAs).ti,ab,kw. |
| 34 | 12 or 13 or 14 or 15 or 16 or 17 or 18 or 19 or 20 or 21 or 22 or 23 or 24 or 25 or 26 or 27 or 28 or 29 or 30 or 31 or 32 or 33 |
| 35 | 11 and 34 |
| 36 | (35 and human/) or (35 not nonhuman/) |
| 37 | 36 not (conference.pt. or medline.cr. or case report/) |
| 38 | limit 37 to (English language) |

**Table S9.** GO terms enriched with genes associated with cleft lip with/without cleft palate (CL/P) in humans

| **GO domain** | **Top 10 enriched terms** | **# CL/P genes** | **FDR** |
| --- | --- | --- | --- |
| Biological Process | palate development | 20 | 7.41 x 10^-18^ |
|  | positive regulation of transcription, DNA-templated | 34 | 1.89 x 10^-13^ |
|  | in utero embryonic development | 19 | 3.71 x 10^-9^ |
|  | positive regulation of transcription from RNA polymerase II promoter | 39 | 9.39 x 10^-9^ |
|  | cell fate commitment | 11 | 9.25 x 10^-8^ |
|  | inner ear morphogenesis | 11 | 3.39 x 10^-7^ |
|  | odontogenesis of dentin-containing tooth | 11 | 6.10 x10^-7^ |
|  | negative regulation of cell proliferation | 23 | 6.68 x 10^-7^ |
|  | branching involved in ureteric bud morphogenesis | 10 | 1.00 x 10^-6^ |
|  | face morphogenesis | 9 | 1.40 x 10^-6^ |
| Molecular Function | frizzled binding | 11 | 4.21 x 10^-9^ |
|  | sequence-specific DNA binding | 23 | 5.04 x 10^-5^ |
|  | growth factor activity | 13 | 2.39 x 10^-4^ |
|  | protein homodimerization activity | 26 | 3.56 x 10^-4^ |
|  | 1-phosphatidylinositol-3-kinase activity | 8 | 4.63 x 10^-4^ |
|  | transcription factor activity, sequence-specific DNA binding | 28 | 0.0052 |
|  | phosphatidylinositol-4,5-bisphosphate 3-kinase activity | 8 | 0.0060 |
|  | protein tyrosine kinase activity | 10 | 0.0182 |
|  | chromatin binding | 16 | 0.0284 |
|  | transcriptional repressor activity, RNA polymerase II transcription regulatory region sequence-specific binding | 7 | 0.0543 |
| Cellular Component | proteinaceous extracellular matrix | 22 | 6.88 x 10^-10^ |
|  | extracellular region | 48 | 3.80 x 10^-8^ |
|  | cell surface | 27 | 7.89 x 10^-8^ |
|  | extracellular space | 36 | 3.73 x 10^-4^ |
|  | endoplasmic reticulum lumen | 11 | 0.0389 |
|  | endocytic vesicle membrane | 7 | 0.0762 |
|  | nucleus | 81 | 0.1215 |
|  | cytosol | 56 | 0.1492 |
|  | extracellular matrix | 12 | 0.3244 |
|  | integral component of plasma membrane | 29 | 0.7665 |

FDR: Benjamini–Hochberg adjustment for multiple test correction[22].

**Table S10.** GO Biological Process terms enriched with human CL/P genes (FDR < 0.005)

| GO Biological Process | CL/P genes (FDR < 0.005) |
| --- | --- |
| palate development | *WNT5A, SATB2, GABRB3, WNT3A, TGFBR1, TGFB3, SMAD2, SKI, VAX1, EPHB3, SHH, TGFB2, EPHB2, SUMO1, CHD7, MSX1, FOXF2, WNT9B, TFAP2A, WNT11* |
| positive regulation of transcription, DNA-templated | *E2F1, WNT5A, BLM, WNT3A, ARNT2, TGFB3, PAX6, FGF10, TP63, CDH1, PAX3, GLI2, SOX9, TGFB1, SHH, FOXF2, BCL3, RARA, WNT6, RUNX2, FGF2, DVL2, BMP4, TGFBR1, SMAD2, TOX3, RFC1, IRF6, FOXE1, TFAP2A, ROR2, WNT11, PTCH1, PHF8* |
| in utero embryonic development | *FGFR2, FGFR1, NOG, TGFBR1, WNT3A, ARNT2, JAG2, TGFB3, GJA1, SMAD2, GLI2, MYH9, TPM1, CHD7, MSX1, TANC2, WNT9B, PTCH1, NOS3* |
| positive regulation of transcription from RNA polymerase II promoter | *E2F1, FGFR2, WNT5A, BACH1, NOG, WNT3A, ARNT2, TGFB3, PAX6, TP63, FGF10, ZEB2, PAX3, GREM1, GLI2, SOX9, SHH, TGFB1, CHD7, PAX9, PAX7, FOXF2, BCL3, RARA, FGF1, FGF2, BMP4, SATB2, MAFB, SKI, SMAD2, EN2, SMAD1, DLX3, HOXB4, EYA1, MSX1, TFAP2A, BMPR1B* |
| cell fate commitment | *FGFR2, WNT5A, SPRY2, WNT10A, WNT3, WNT5B, WNT9B, ROR2, SMAD2, WNT11, WNT6* |
| inner ear morphogenesis | *FGFR2, SPRY2, FGFR1, CHD7, MAFB, WNT3A, TFAP2A, ROR2, NTN1, FZD6, EPHB2* |
| odontogenesis of dentin-containing tooth | *DLX3, BMP4, DLX1, MSX1, JAG2, TP63, FGF10, GLI2, WNT6, RUNX2, SHH* |
| negative regulation of cell proliferation | *BMP4, COL4A3, JARID2, TGFB3, FGF10, BRIP1, SMAD2, SKI, SMAD1, TIMP2, TGFB1, TGFB2, MSX2, SPRY2, MSX1, IRF6, PEMT, ROR2, TFAP2A, NOS3, RARA, AXIN2, FGF2* |
| branching involved in ureteric bud morphogenesis | *BMP4, EYA1, BCL2, WNT9B, PTCH1, GREM1, WNT6, SOX9, FGF2, SHH* |
| face morphogenesis | *NOG, MSX1, PAX9, CRISPLD2, TGFB3, SKI, MMP2, TGFB1, TGFB2* |
| negative regulation of transcription from RNA polymerase II promoter | *E2F1, FGFR2, BACH1, FGFR1, NOG, TBX22, PAX6, TP63, ZEB2, GLI2, TGFB1, SHH, MSX2, RARA, BMP4, SATB2, JARID2, VAX2, SKI, SMAD2, VAX1, FOXP2, HOXB3, HOXB4, DLX1, MSX1, RFC1, FOXE1, TFAP2A, PTCH1* |
| positive regulation of cell division | *FGFR2, TGFB3, TGFA, PDGFC, FGF1, FGF2, TGFB1, FGF3, SHH, TGFB2* |
| epithelial to mesenchymal transition | *FGFR2, WNT5A, NOG, TGFBR1, FOXF2, WNT11, SOX9, TGFB1, TGFB2* |
| negative regulation of apoptotic process | *WNT5A, BMP4, ADAMTS20, MMP9, ARNT2, TP63, SOX9, GREM1, GLI2, SHH, MSX2, SPRY2, BAG4, MSX1, BCL2, PAX7, TGFA, BCL3, TFAP2A, WNT11, RARA, GSTP1, TEX11* |
| neural tube closure | *DVL2, MTHFD1, BMP4, NOG, PTCH1, RARA, SKI, TGFB1, MTHFD1L, TGFB2, FZD6* |
| hair follicle morphogenesis | *FGFR2, WNT10A, BCL2, FOXE1, TP63, FGF10, SHH, TGFB2* |
| axon guidance | *WNT5A, RYK, EFNB1, WNT3A, NECTIN1, PAX6, VAX1, GLI2, EPHB3, NTN1, SHH, TGFB2, EPHB2, WNT3* |
| folic acid metabolic process | *MTHFD1, MTHFR, DHFR, FOLR1, SLC19A1, MTHFD1L, MTRR* |
| skeletal system development | *FGFR1, NOG, FGFR3, EVC, TGFBR1, MMP9, JAG2, TP63, GLI2, SOX9, TGFB2, CHD7, BMPR1B* |
| smoothened signaling pathway | *BMP4, EVC2, EVC, PAX6, ROR2, PTCH1, HHIP, GLI2, CENPJ, SHH* |
| cellular response to retinoic acid | *WNT5A, WNT3, WNT5B, WNT3A, SLC6A4, WNT9B, RARA, WNT11, WNT6, SOX9* |
| negative regulation of transcription, DNA-templated | *WNT5A, E2F1, BMP4, JARID2, TBX22, TP63, SMAD2, SOX9, GREM1, TGFB1, FOXP2, MSX2, SUMO1, PAX9, FOXF2, FOXG1, FOXE1, BCL3, TFAP2A, RARA, WNT11, RUNX2* |
| embryonic pattern specification | *FGFR2, SATB2, EFNB1, FGF10, SMAD2, SMAD1, SHH* |
| lung development | *FGFR2, WNT5A, FGF18, CRISPLD2, SMAD2, NOS3, GLI2, FGF1, FGF2, SHH* |
| positive regulation of cell proliferation | *FGFR2, FGFR1, FGF18, FGFR3, WNT3A, TGFBR1, ARNT2, SOX9, GREM1, NTN1, SHH, TGFB1, TGFB2, BCL2, TGFA, PDGFC, RARA, FGF1, FGF2, RUNX2, FGF3* |
| positive regulation of gene expression | *E2F1, WNT10A, NOG, TGFBR1, WNT3A, SLC6A4, PAX6, GJA1, SMAD1, TGFB1, TGFB2, SPRY2, WNT3, TFAP2A, WNT11, WNT6* |
| methionine biosynthetic process | *MTHFD1, BHMT2, MTR, BHMT, MTRR* |
| wound healing | *WNT5A, NOG, WNT5B, TGFBR1, TGFB3, TGFA, FGF10, FGF2, TPM1, TGFB2* |
| response to drug | *ASS1, CYP1A1, SLC6A4, CDH1, ABCB1, CYP2E1, TIMP2, TGFB1, RAD51, TGFB2, TYMS, MTHFR, BCL2, PEMT, TGFA, PTCH1, GAD1* |
| cartilage development | *HOXB3, WNT5A, TYMS, NOG, SATB2, EVC, PAX7, SMAD1, SOX9* |
| positive regulation of epithelial cell proliferation | *FGFR2, BMP4, EYA1, NOG, TGFA, FGF10, FGF1, SOX9, TGFB1* |
| thymus development | *JARID2, MAFB, TGFBR1, BCL2, FOXE1, FGF10, EPHB3, SHH* |
| odontogenesis | *FGFR2, BMP4, WNT10A, PAX9, TGFB3, AXIN2, TGFB2* |
| pituitary gland development | *BMP4, NOG, MSX1, PAX6, FGF10, CDH1, GLI2* |
| neuron fate commitment | *BMP4, TGFBR1, PAX7, PAX6, SHH, TGFB2* |
| embryonic cranial skeleton morphogenesis | *FGFR2, BMP4, TGFBR1, WNT9B, TFAP2A, SMAD2, RUNX2* |
| peptidyl-tyrosine phosphorylation | *FGFR2, FGFR1, FGF18, FGFR3, RYK, ROR2, FGF10, FGF1, EPHB3, FGF2, FGF3, EPHB2* |
| neuron differentiation | *WNT5A, WNT10A, WNT3, WNT5B, RYK, WNT3A, WNT9B, WNT11, WNT6, RUNX2* |
| phosphatidylinositol-3-phosphate biosynthetic process | *FGFR2, FGFR1, FGF18, FGFR3, FGF10, FGF1, FGF2, FGF3* |
| angiogenesis | *FGFR2, FGFR1, FGF18, COL4A2, FGF10, EPHB3, MYH9, MMP2, TGFB2, EPHB2, HOXB3, TGFA, NOS3, FGF1* |
| negative regulation of epithelial cell proliferation | *FGFR2, WNT5A, BMP4, PAX6, PTCH1, SOX9, TGFB1, TGFB2* |
| limb bud formation | *FGFR2, WNT3, FGF10, SOX9, SHH* |
| fibroblast growth factor receptor signaling pathway | *FGFR2, FGFR1, FGF18, FGFR3, FGF10, FGF12, FGF1, FGF2, FGF3* |
| ureteric bud development | *FGFR2, BMP4, FGFR1, SMAD2, RARA, SMAD1, TGFB1* |
| chondrocyte differentiation | *BMP4, FGFR1, FGFR3, WNT5B, GLI2, RUNX2, TGFB1* |
| positive regulation of osteoblast differentiation | *MSX2, BMP4, TP63, GJA1, SMAD1, BMPR1B, RUNX2, FGF2* |
| outflow tract septum morphogenesis | *FGFR2, MSX2, BMP4, RARA, TGFB2, PARVA* |
| embryonic limb morphogenesis | *FGFR1, TP63, PTCH1, SKI, GREM1, SHH, TGFB2* |
| SMAD protein signal transduction | *BMP4, TGFB3, ROR2, SMAD2, SKI, SMAD1, TGFB1, TGFB2* |
| prostatic bud formation | *NOG, TP63, FGF10, GLI2* |
| organ induction | *BMP4, FGFR1, FGF10, FGF1, FGF2* |
| Wnt signaling pathway | *WNT5A, DVL2, WNT10A, WNT3, WNT5B, RYK, WNT3A, WNT9B, VAX2, WNT11, AXIN2, WNT6* |
| positive regulation of mesenchymal cell proliferation | *FGFR2, WNT5A, FGFR1, TP63, SOX9, FOXP2* |
| embryonic hindlimb morphogenesis | *MSX2, BMP4, CHD7, MSX1, WNT3, SHH* |
| negative regulation of canonical Wnt signaling pathway | *WNT5A, DVL2, NOG, WNT5B, ROR2, WNT11, GREM1, AXIN2, SOX9, SHH, FZD6* |
| somite development | *MTHFD1, NOG, PTCH1, WNT11, SHH* |
| multicellular organism development | *WNT5A, WNT10A, WNT5B, TBX22, PAX6, EN2, SHH, FZD6, HOXB3, CLPTM1, REG3A, DCAF7, PKP1, WNT9B, ROR2, WNT11, WNT6, FGF1, FGF3* |
| embryonic skeletal system development | *WNT5A, NOG, DLX1, PAX7, WNT11, SHH* |
| midbrain development | *FGFR2, FGFR1, MSX1, SMAD1, EN2, SHH* |
| regulation of phosphatidylinositol 3-kinase signaling | *FGFR2, FGFR1, FGF18, FGFR3, FGF10, FGF1, FGF2, FGF3* |
| positive regulation of cartilage development | *WNT5A, BMP4, SMAD1, BMPR1B, SOX9* |
| embryonic forelimb morphogenesis | *MSX2, MSX1, WNT3, TFAP2A, RUNX2, SHH* |
| negative regulation of cartilage development | *NOG, RARA, WNT11, TGFB2* |
| BMP signaling pathway involved in heart development | *MSX2, BMP4, NOG, MSX1* |
| positive regulation of epithelial to mesenchymal transition | *TGFBR1, TGFB3, SMAD2, AXIN2, TGFB1, TGFB2* |
| osteoblast development | *MSX2, SATB2, GLI2, RUNX2, SHH* |
| negative regulation of chondrocyte differentiation | *BMP4, TGFBR1, GLI2, GREM1, SOX9* |
| embryonic digit morphogenesis | *WNT5A, BMP4, NOG, ROR2, GJA1, GLI2, SHH* |
| canonical Wnt signaling pathway | *WNT5A, DVL2, WNT3, RYK, WNT3A, WNT9B, WNT11, SHH* |
| retina development in camera-type eye | *CHD7, NECTIN1, PAX6, VAX2, SKI, BMPR1B, SOX9* |
| pharyngeal arch artery morphogenesis | *BMP4, NOG, FOLR1, TGFB2* |
| salivary gland morphogenesis | *TGFB3, PAX6, TGFB1, TGFB2* |
| bud elongation involved in lung branching | *FGFR2, BMP4, SPRY2, FGF10* |
| S-adenosylmethionine metabolic process | *BHMT2, MTHFR, BHMT, PEMT* |
| lacrimal gland development | *FGFR2, PAX6, FGF10, SOX9* |
| extracellular matrix organization | *COL4A4, COL4A3, COL4A2, CRISPLD2, ADAMTS20, WNT3A, FOXF2, BCL3, CDH1, SOX9, FGF2* |
| response to estradiol | *ASS1, ARNT2, SLC6A4, FGF10, PTCH1, RARA, TGFB1, GSTP1* |
| positive regulation of phospholipase activity | *FGFR2, FGFR1, FGFR3, APOC2* |
| collagen catabolic process | *COL4A4, COL4A3, COL4A2, MMP9, MMP3, MMP2, MMP1* |
| cobalamin metabolic process | *MTR, PRSS1, TCN2, TCN1, MTRR* |
| phosphatidylinositol phosphorylation | *FGFR2, FGFR1, FGF18, FGFR3, FGF10, FGF1, FGF2, FGF3* |
| cochlea morphogenesis | *WNT5A, DVL2, EYA1, GLI2, SOX9* |
| cellular protein localization | *WNT5A, DVL2, EYA1, DMD, WNT3A, AXIN2* |
| sensory perception of sound | *SPRY2, COL4A3, FGFR1, EYA1, CHD7, GABRB3, TFAP2A, MYH14, PAX3* |
| positive regulation of ERK1 and ERK2 cascade | *FGFR2, BMP4, SPRY2, FGF18, FGFR3, FGF10, PDGFC, FGF1, FGF2, TGFB1* |
| osteoblast differentiation | *MSX2, BMP4, NOG, WNT3A, GJA1, WNT11, GLI2, RUNX2* |
| membranous septum morphogenesis | *FGFR2, BMP4, NOG, TGFB2* |
| lung-associated mesenchyme development | *FGFR2, FGFR1, WNT11, SHH* |
| tetrahydrofolate interconversion | *MTHFD1, TYMS, MTHFR, MTHFD1L* |
| phosphatidylinositol-mediated signaling | *FGFR2, FGFR1, FGF18, FGFR3, FGF10, FGF1, FGF2, FGF3* |
| transcription from RNA polymerase II promoter | *BACH1, DVL2, MAFB, ARNT2, PAX6, TP63, SMAD2, PAX3, SMAD1, GLI2, SOX9, DLX3, MSX1, PAX9, FOXF2, TFAP2A, RUNX2* |
| extracellular matrix disassembly | *MMP9, PRSS1, CDH1, TIMP2, MMP3, MMP2, MMP1* |
| BMP signaling pathway | *BMP4, NOG, ROR2, SKI, SMAD1, BMPR1B, RUNX2* |
| cellular response to transforming growth factor beta stimulus | *WNT5A, WNT10A, COL4A2, TGFBR1, SOX9, TGFB1* |
| bone morphogenesis | *FGFR2, MSX1, FGFR3, TFAP2A, SKI* |
| dorsal/ventral neural tube patterning | *BMP4, WNT3A, PAX7, SHH* |
| sulfur amino acid metabolic process | *BHMT2, MTR, BHMT, MTRR* |
| anterior/posterior pattern specification | *HOXB3, MSX2, HOXB4, MSX1, TGFBR1, SMAD2, GLI2* |
| keratinocyte proliferation | *IRF6, TP63, FGF10, PTCH1* |
| dorsal/ventral axis specification | *WNT3, PAX6, VAX2, AXIN2* |

**Table S11.** GO Molecular Function terms enriched with human CL/P genes (FDR<0.005)

| GO Molecular Function | CL/P genes (FDR < 0.005) |
| --- | --- |
| frizzled binding | *WNT5A, DVL2, WNT10A, WNT3, WNT5B, RYK, WNT3A, WNT9B, ROR2, WNT11, WNT6* |
| sequence-specific DNA binding | *BACH1, E2F1, SATB2, PAX6, TP63, EN2, PAX3, GLI2, FOXP2, HOXB3, DLX3, MSX2, HOXB4, DLX1, MSX1, RFC1, BCL2, PAX7, FOXF2, FOXG1, FOXE1, TFAP2A, RARA* |
| growth factor activity | *BMP4, FGF18, JAG2, TGFB3, FGF10, FGF12, TGFB1, TGFB2, TGFA, PDGFC, FGF1, FGF2, FGF3* |
| protein homodimerization activity | *PVR, FGFR2, FGFR1, NOG, SLC6A4, APOC2, TGFB1, MTHFD1L, TGFB2, GCH1, GSTM1, TYMS, BCL2, PCYT1A, PDGFC, NECTIN1, NECTIN2, NECTIN3, SMAD2, SMAD1, MID1, MYH9, TOX3, FOXP2, TFAP2A, CBS* |
| 1-phosphatidylinositol-3-kinase activity | *FGFR2, FGFR1, FGF18, FGFR3, FGF10, FGF1, FGF2, FGF3* |
| transcription factor activity, sequence-specific DNA binding | *E2F1, WNT5A, BACH1, TBX22, ARNT2, PAX6, TP63, PAX3, SOX9, GLI2, PAX9, PAX7, FOXF2, BCL3, RARA, RUNX2, TBX10, SMAD2, VAX2, SMAD1, FOXP2, HOXB3, DLX3, HOXB4, TULP4, IRF6, FOXE1, TFAP2A* |
| phosphatidylinositol-4,5-bisphosphate 3-kinase activity | *FGFR2, FGFR1, FGF18, FGFR3, FGF10, FGF1, FGF2, FGF3* |
| protein tyrosine kinase activity | *FGFR2, FGFR1, FGF18, FGFR3, RYK, FGF10, FGF1, FGF2, FGF3, EPHB2* |
| chromatin binding | *SATB2, JARID2, PAX6, TP63, SMAD2, SKI, GLI2, SOX9, TOX3, RAD51, DLX3, DLX1, CHD7, TFAP2A, PHF8, RUNX2* |
| protein domain specific binding | *WNT5A, DVL2, WNT3, RFC1, WNT3A, RARA, SKI, MYH9, RUNX2, CENPJ* |
| protein heterodimerization activity | *ARNT2, NECTIN1, TGFB3, NECTIN2, NECTIN3, SMAD2, SMAD1, MID1, SOX9, TGFB1, TGFB2, FOXP2, BCL2, RARA, GAD1* |
| protein binding | *PVR, NOG, WNT3A, MMP9, SLC6A4, ARNT2, TGFB3, JAG2, FGF10, FGF12, MMP3, MMP2, SHH, TGFB1, TGFB2, VCL, REG3A, WNT3, PDGFC, RARA, EIF2B3, SATB2, CYP1A1, EFNB1, MYH9, TOX3, TNS1, DCAF7, RFC1, FOXG1, TFAP2A, ROR2, FGFR2, WNT5A, FGFR1, FGFR3, ASS1, BLM, TIMP2, GREM1, SOX9, ASL, EPHB2, DMD, AXIN2, RUNX2, GAD1, HECTD1, DVL2, BMP4, MAFB, RYK, TGFBR1, BRIP1, ABCB1, SMAD2, SKI, SMAD1, NTN1, FOXP2, SYNE3, PKP1, MTR, PHF8, E2F1, BACH1, TBX22, PAX6, TP63, GJA1, ZEB2, PAX3, GLI2, MTHFD1, BAG4, SPRY2, KISS1R, PAX9, TGFA, NOS3, YOD1, HHIP, FGF1, FGF2, FGF3, KIF2A, NECTIN1, NECTIN2, NECTIN3, MTRR, RAD51, EYA1, WNT11, GSTP1, PARVA, CDH1, TPM1, GCH1, MSX2, SUMO1, CHD7, KIF7, BCL2, SEC16A, BCL3, COL4A3, COL4A2, NAT2, MID1, CENPJ, FZD6, CLPTM1, IRF6, PTCH1, BMPR1B, CBS, TEX11* |
| receptor agonist activity | *WNT5A, WNT3, WNT3A, GREM1* |
| type III transforming growth factor beta receptor binding | *TGFB3, TGFB1, TGFB2* |
| fibroblast growth factor-activated receptor activity | *FGFR2, FGFR1, FGFR3* |
| fibroblast growth factor receptor binding | *FGF10, FGF1, FGF2, FGF3* |
| RNA polymerase II transcription factor activity, sequence-specific DNA binding | *ARNT2, FOXG1, PAX6, FOXE1, TFAP2A, SOX9, RUNX2, FOXP2* |
| RNA polymerase II core promoter sequence-specific DNA binding | *HOXB3, PAX6, TFAP2A, SMAD1, RUNX2* |
| transcription factor binding | *E2F1, MSX2, SUMO1, MAFB, BCL2, FOXF2, PAX6, BCL3, SMAD2, RARA* |

**Table S12.** GO Cellular Component terms enriched with human CL/P genes (FDR < 0.05)

| GO Cellular Component | CL/P genes (FDR < 0.05) |
| --- | --- |
| proteinaceous extracellular matrix | *COL4A4, WNT5A, BMP4, COL4A3, WNT10A, WNT5B, COL21A1, ADAMTS20, WNT3A, MMP9, MMP3, TIMP2, MMP2, MMP1, SHH, TGFB1, WNT3, CRISPLD2, WNT9B, WNT11, WNT6, FGF1* |
| extracellular region | *FGFR2, WNT5A, FGFR1, FGF18, NOG, WNT5B, FGFR3, COL21A1, WNT3A, MMP9, F13A1, TGFB3, PRSS1, APOC2, FGF10, CDH1, MMP3, TIMP2, EPHB3, MMP2, MMP1, SHH, TGFB1, TGFB2, VCL, EPHB2, WNT3, CRISPLD2, REN, PDGFC, HHIP, FGF1, WNT6, PRSS35, FGF2, FGF3, BMP4, COL4A4, WNT10A, COL4A3, COL4A2, NECTIN1, HLA-C, TCN2, TCN1, NTN1, WNT9B, WNT11* |
| cell surface | *PVR, WNT5A, FGFR2, FGFR3, WNT5B, WNT3A, TGFB3, FGF10, TIMP2, GREM1, TGFB1, SHH, KISS1R, FOLR1, DMD, TGFA, RARA, PDGFC, HHIP, WNT6, CR1, TGFBR1, NECTIN2, ABCB1, HLA-C, FZD6, TNS1* |
| extracellular space | *PVR, WNT5A, FGF18, NOG, WNT5B, WNT3A, MMP9, TGFB3, FGF10, APOC2, FGF12, MMP3, GREM1, TIMP2, MMP2, TGFB1, SHH, TGFB2, REG3A, WNT3, SERPINA6, REN, TGFA, PDGFC, FGF1, WNT6, FGF2, BMP4, WNT10A, MROH7, ADAMTS20, TCN2, TCN1, WNT9B, WNT11, GSTP1* |
| endoplasmic reticulum lumen | *COL4A4, WNT5A, COL4A3, COL4A2, WNT3, WNT5B, COL21A1, WNT3A, PDGFC, WNT6, SHH* |
| endocytic vesicle membrane | *WNT5A, WNT3, WNT5B, WNT3A, PTCH1, NOS3, WNT6* |
| nucleus | *BACH1, E2F1, TBX22, ARNT2, TGFB3, PAX6, TP63, FGF10, ZEB2, FGF12, GLI2, MMP2, TGFB1, SPRY2, BAG4, PAX9, PAX7, FOXF2, TGFA, PDGFC, NOS3, RARA, YOD1, FGF2, KIF2A, SATB2, TBX10, EFNB1, VAX2, VAX1, MYH9, TOX3, ESCO2, RAD51, EVC2, EYA1, MSX1, RFC1, FOXG1, TFAP2A, GSTP1, PARVA, FGFR2, FGFR1, FGFR3, ASS1, BLM, SOX9, GCH1, MSX2, TYMS, SUMO1, CHD7, FOLR1, BCL2, DMD, BCL3, AXIN2, RUNX2, HECTD1, DVL2, RECQL5, JARID2, MAFB, RYK, BRIP1, SMAD2, SKI, EN2, SMAD1, FOXP2, HOXB3, DLX3, HOXB4, DLX1, PKP1, IRF6, FOXE1, ZNF385B, PHF8, CBS* |
| cytosol | *BACH1, SLC6A4, ADH1C, GJA1, TP63, GLI2, MTHFD1L, SHH, VCL, MTHFD1, GSTM1, BAG4, SPRY2, PDGFC, NOS3, YOD1, FGF1, EIF2B3, KIF2A, GSTT1, ARHGAP29, MYH9, MTRR, BHMT, GSTP1, PARVA, TPH2, FGFR1, ASS1, RHPN2, PAH, EPHB3, ASL, TPM1, EPHB2, GCH1, TYMS, MTHFR, BCL2, DMD, SEC16A, PCYT1A, AXIN2, DVL2, BHMT2, NAT1, NAT2, SMAD2, SMAD1, MID1, CENPJ, DHFR, IRF6, MTR, MYH14, CBS* |
| extracellular matrix | *FGFR2, COL4A2, COL21A1, ADAMTS20, TGFB3, FGF10, TIMP2, MYH9, MMP2, TGFB1, MMP1, TGFB2* |
| integral component of plasma membrane | *PVR, FGFR2, FGFR1, FGFR3, GABRB3, SLC6A4, JAG2, GJA1, AQP7, ABCA4, EPHB3, SLC19A1, EPHB2, KISS1R, FOLR1, TGFA, HHIP, CR1, RYK, EFNB1, NECTIN1, NECTIN2, NECTIN3, HLA-C, FZD6, CLPTM1, SLC25A13, ROR2, BMPR1B* |
| transcription factor complex | *SATB2, MAFB, ARNT2, FOXF2, TP63, SMAD2, SKI, SMAD1, RUNX2* |
| collagen type IV trimer | *COL4A4, COL4A3, COL4A2* |
| Golgi lumen | *WNT5A, WNT3, WNT5B, WNT3A, WNT6, TGFB1* |

**Table S13.** Top 30 Human Phenotype Ontology Categories

| **Rank** | **Description** | **Genes in category** | **Overlap** | **FDR** |
| --- | --- | --- | --- | --- |
| 1 | Abnormal number of teeth | 229 | 28 | 1.66x10^-10^ |
| 2 | Cleft lip | 151 | 23 | 2.25 x10^-10^ |
| 3 | Cleft upper lip | 139 | 22 | 2.52 x10^-10^ |
| 4 | Reduced number of teeth | 201 | 25 | 9.22 x10^-10^ |
| 5 | Oral cleft | 468 | 36 | 5.45 x10^-9^ |
| 6 | Conical tooth | 25 | 10 | 4.66 x10^-8^ |
| 7 | Cleft palate | 438 | 33 | 7.36 x10^-8^ |
| 8 | Abnormal hard palate morphology | 439 | 33 | 7.36 x10^-8^ |
| 9 | Oligodontia | 38 | 11 | 1.87 x10^-7^ |
| 10 | Microdontia | 101 | 16 | 2.34 x10^-7^ |
| 11 | Abnormality of dental morphology | 173 | 20 | 3.83 x10^-7^ |
| 12 | Non-midline cleft lip | 27 | 9 | 1.53 x10^-6^ |
| 13 | Clinodactyly of the 5th finger | 237 | 22 | 2.80 x10^-6^ |
| 14 | Deviation of the 5th finger | 239 | 22 | 3.04 x10^-6^ |
| 15 | Abnormality of the maxilla | 112 | 15 | 6.33 x10^-6^ |
| 16 | Hypoplasia of the maxilla | 99 | 14 | 7.66 x10^-6^ |
| 17 | Finger syndactyly | 170 | 18 | 7.66 x10^-6^ |
| 18 | Triangular shaped phalanges of the hand | 132 | 16 | 7.66 x10^-6^ |
| 19 | Finger clinodactyly | 255 | 22 | 7.66 x10^-6^ |
| 20 | Clinodactyly | 339 | 25 | 1.59 x10^-5^ |
| 21 | Deviation of the hand or of fingers of the hand | 341 | 25 | 1.70 x10^-5^ |
| 22 | Abnormality of thumb phalanx | 128 | 15 | 2.70 x10^-5^ |
| 23 | Deviation of finger | 328 | 24 | 3.17 x10^-5^ |
| 24 | Duplication of phalanx of hand | 173 | 17 | 4.48 x10^-5^ |
| 25 | Duplication of hand bones | 174 | 17 | 4.50 x10^-5^ |
| 26 | Duplication of bones involving the upper extremities | 174 | 17 | 4.50 x10^-5^ |
| 27 | Synostosis of carpal bones | 22 | 7 | 5.69 x10^-5^ |
| 28 | Aplasia/Hypoplasia of fingers | 221 | 19 | 5.69 x10^-5^ |
| 29 | Abnormality of the nail | 344 | 24 | 6.21 x10^-5^ |
| 30 | Abnormality of the incisor | 44 | 9 | 6.36 x10^-5^ |

**Table S14.** KEGG pathways enriched with genes associated with cleft lip with/without cleft palate (CL/P) in humans

| **Pathway name** | **Candidate genes in the pathway** | **# CL/P genes** | **FDR** |
| --- | --- | --- | --- |
| Pathways in cancer | *E2F1*, *FGFR2*, *WNT5A*, *FGFR1*, *FGF18*, *WNT5B*, *FGFR3*, *WNT3A*, *MMP9*, *ARNT2*, *TGFB3*, *FGF10*, *CDH1*, *FGF12*, *GLI2*, *MMP2*, *MMP1*, *SHH*, *TGFB1*, *TGFB2*, *WNT3*, *BCL2*, *TGFA*, *RARA*, *HHIP*, *AXIN2*, *WNT6*, *FGF1*, *FGF2*, *FGF3*, *DVL2*, *BMP4*, *COL4A4*, *WNT10A*, *COL4A3*, *COL4A2*, *TGFBR1*, *SMAD2*, *RAD51*, *FZD6*, *WNT9B*, *WNT11*, *PTCH1* | 43 | 3.04 x 10^-19^ |
| Basal cell carcinoma | *WNT5A*, *BMP4*, *DVL2*, *WNT10A*, *WNT5B*, *WNT3A*, *GLI2*, *SHH*, *FZD6*, *WNT3*, *WNT9B*, *WNT11*, *PTCH1*, *HHIP*, *AXIN2*, *WNT6* | 16 | 2.41 x 10^-11^ |
| Hippo signaling pathway | *WNT5A*, *DVL2*, *BMP4*, *WNT10A*, *WNT5B*, *TGFBR1*, *WNT3A*, *TGFB3*, *CDH1*, *SMAD2*, *SMAD1*, *GLI2*, *TGFB1*, *FZD6*, *TGFB2*, *WNT3*, *WNT9B*, *WNT11*, *BMPR1B*, *AXIN2*, *FGF1*, *WNT6* | 22 | 2.08 x 10^-10^ |
| Signaling pathways regulating pluripotency of stem cells | *WNT5A*, *DVL2*, *BMP4*, *FGFR2*, *WNT10A*, *FGFR1*, *WNT5B*, *FGFR3*, *JARID2*, *WNT3A*, *PAX6*, *SMAD2*, *SMAD1*, *FZD6*, *WNT3*, *WNT9B*, *WNT11*, *BMPR1B*, *AXIN2*, *WNT6*, *FGF2* | 21 | 4.95 x 10^-10^ |
| HTLV-I infection | *WNT5A*, *DVL2*, *E2F1*, *WNT10A*, *WNT5B*, *TGFBR1*, *WNT3A*, *TGFB3*, *HLA-C*, *SMAD2*, *TGFB1*, *FZD6*, *TGFB2*, *MSX2*, *MSX1*, *WNT3*, *WNT9B*, *WNT11*, *WNT6* | 19 | 7.84 x 10^-4^ |
| Proteoglycans in cancer | *WNT5A*, *WNT10A*, *FGFR1*, *WNT5B*, *MMP9*, *WNT3A*, *MMP2*, *TGFB1*, *FZD6*, *TGFB2*, *WNT3*, *WNT9B*, *WNT11*, *PTCH1*, *WNT6*, *FGF2* | 16 | 0.0033 |
| Melanoma | *E2F1*, *FGFR1*, *FGF18*, *FGF10*, *CDH1*, *PDGFC*, *FGF12*, *FGF1*, *FGF2*, *FGF3* | 10 | 0.0054 |
| One carbon pool by folate | *MTHFD1*, *TYMS*, *MTHFR*, *DHFR*, *MTR*, *MTHFD1L* | 6 | 0.0269 |
| Melanogenesis | *WNT5A*, *DVL2*, *WNT10A*, *WNT3*, *WNT5B*, *WNT3A*, *WNT9B*, *WNT11*, *WNT6*, *FZD6* | 10 | 0.0891 |
| Chemical carcinogenesis | *GSTM1*, *CYP1A1*, *NAT1*, *NAT2*, *ADH1C*, *GSTT1*, *EPHX1*, *CYP2E1*, *GSTP1* | 9 | 0.1075 |
| TGF-beta signaling pathway | *BMP4*, *NOG*, *TGFBR1*, *TGFB3*, *SMAD2*, *SMAD1*, *BMPR1B*, *TGFB1*, *TGFB2* | 9 | 0.1522 |
| Pancreatic cancer | *E2F1*, *TGFBR1*, *TGFB3*, *TGFA*, *SMAD2*, *TGFB1*, *RAD51*, *TGFB2* | 8 | 0.1880 |
| Wnt signaling pathway | *WNT5A*, *DVL2*, *WNT10A*, *WNT3*, *WNT5B*, *WNT3A*, *WNT9B*, *WNT11*, *AXIN2*, *WNT6*, *FZD6* | 11 | 0.2204 |
| Adherens junction | *FGFR1*, *TGFBR1*, *NECTIN1*, *NECTIN2*, *CDH1*, *SMAD2*, *NECTIN3*, *VCL* | 8 | 0.3281 |
| Bladder cancer | *E2F1*, *FGFR3*, *MMP9*, *CDH1*, *MMP2*, *MMP1* | 6 | 0.9540 |
| Colorectal cancer | *TGFBR1*, *BCL2*, *TGFB3*, *SMAD2*, *AXIN2*, *TGFB1*, *TGFB2* | 7 | 1.0008 |
| Metabolism of xenobiotics by cytochrome P450 | *GSTM1*, *CYP1A1*, *ADH1C*, *GSTT1*, *EPHX1*, *CYP2E1*, *GSTP1* | 7 | 2.5110 |
| MAPK signaling pathway | *FGFR2*, *FGF18*, *FGFR1*, *FGFR3*, *TGFBR1*, *TGFB3*, *FGF10*, *FGF12*, *FGF1*, *FGF2*, *FGF3*, *TGFB1*, *TGFB2* | 13 | 2.5353 |
| PI3K-Akt signaling pathway | *COL4A4*, *FGFR2*, *COL4A3*, *FGFR1*, *FGF18*, *COL4A2*, *FGFR3*, *FGF10*, *FGF12*, *BCL2*, *NOS3*, *PDGFC*, *FGF1*, *FGF2*, *FGF3* | 15 | 4.2970 |

FDR is obtained by Benjamini–Hochberg adjustment for multiple test correction[22].
