# Supplementary material for: MicroRNA-655-3p and microRNA-497-5p inhibit cell proliferation in cultured human lip cells through the regulation of genes related to human cleft lip
Source: BMC Med Genomics. 2019 May 23;12:70. doi: 10.1186/s12920-019-0535-2 (PMC6533741; doi:10.1186/s12920-019-0535-2)
Supplement: Supplementary file 1 — Table S1. PCR primer sets used in this study. Table S2. Summary of databases searched. Table S3. Genes with significant contribution to human CL/P (identified through single gene studies). Table S4. Genes with significant contribution to human CL/P (identified through multiple genes studies). Table S5. Genes with significant contribution to human CL/P (unknown coding genes). Table S6. Genes without significant contribution to human CL/P. Table S7. CL/P candidate genes with significant signals in GWAS. Table S8. CL/P candidate genes without significant signals in GWAS. Table S9. GO terms enriched with genes associated with cleft lip with/without cleft palate (CL/P) in humans. Table S10. GO Biological Process terms enriched with human CL/P genes (FDR < 0.005). Table S11. GO Molecular Function terms enriched with human CL/P genes (FDR < 0.005). Table S12. GO Cellular Component terms enriched with human CL/P genes (FDR < 0.05). Table S13. Top 30 Human Phenotype Ontology Categories. Table S14. KEGG pathways enriched with genes associated with cleft lip with/without cleft palate (CL/P) in humans. (ZIP 292 kb) [file 12920_2019_535_MOESM1_ESM.zip › 9 Human Cleft Lip_Supplemental ReferencesR2.docx]

**Supplemental References**

1. Khanna, R., Tikku, T., Jain, G. S., Srivastava, K., Darokar, M. P., & Mishra, R. K. (2014). Sequencing of the interferon regulatory factor 6 (IRF6) gene and correlation to its phenotypes in familial non-syndromic cleft lip and palate in North Indian population. *European Journal of Plastic Surgery*, *37*(6), 319-326. No PMID
2. Wu W, Hao J, Hua L, Li F, Chen Y, Liu J. (2016). Association of polymorphisms of IRF6 to non-syndromic cleft lip with or without palate in Guangdong population. Int J Clin Exp Med, 9(6):11732-11739. No PMID
3. Khanna, R., Tikku, T., Jain, G. S., Srivastava, K., Darokar, M. P., & Mishra, R. K. (2014). Sequencing of the interferon regulatory factor 6 (IRF6) gene and correlation to its phenotypes in familial non-syndromic cleft lip and palate in North Indian population. *European Journal of Plastic Surgery*, *37*(6), 319-326. No PMID
4. Sözen, M. A., Tolarova, M. M., & Spritz, R. A. (2009). Study Of The CLPTM1 Gene In South American Non-Syndromic Cleft Lip Patients With Or Without Palate. No PMID
5. Sözen, M. A., Hecht, J. T., & Spritz, R. A. (2008). Lack of mutations in the PVRL3 gene in North American caucasians with non-syndromic cleft lip/palate. *Genetics and Molecular Biology*, *31*(3), 649-650. No PMID
6. Suazo, J., Santos, J. L., Jara, L., & Blanco, R. (2008). Assessment of the association between SMAD1 and HHIP gene variation and non-syndromic cleft-lip palate in Chilean case-parent trios. *Genetics and Molecular Biology*, *31*(3), 639-642. No PMID
7. Shen, Y., Cui, Y., Wan, W., Zhou, X., Cheng, L., Lu, Z., & Liu, J. (2009). Association of Single Nucleotide Polymorphisms in IRF6 and TGFA Genes With Nonsyndromic Cleft Lip With Or Without Cleft Palate in Chinese Patients. *Journal of Nanjing Medical University*, *23*(1), 40-45. No PMID
